# Extracellular vesicles as therapeutic tools in regenerative dentistry

**DOI:** 10.1186/s13287-024-03936-5

**Published:** 2024-10-14

**Authors:** Evelyn Jingwen Xia, Shasha Zou, Xiu Zhao, Wei Liu, Yang Zhang, Irene Shuping Zhao

**Affiliations:** 1 School of Dentistry, Shenzhen University Medical School, 1088 Xueyuan Ave, Shenzhen, 518015 China; 2 Longgang Center for Chronic Disease Control, Shenzhen, 518172 China; 3 Department of Stomatology, Shenzhen University General Hospital, Shenzhen, 518015 China; 4 School of Biomedical Engineering, Shenzhen University Medical School, Shenzhen, 518015 China

**Keywords:** Extracellular vesicles, Exosomes, Dentistry, Dental tissue, Tissue regeneration

## Abstract

Dental and maxillofacial diseases are always accompanied by complicated hard and soft tissue defects, involving bone, teeth, blood vessels and nerves, which are difficult to repair and severely affect the life quality of patients. Recently, extracellular vesicles (EVs) secreted by all types of cells and extracted from body fluids have gained more attention as potential solutions for tissue regeneration due to their special physiological characteristics and intrinsic signaling molecules. Compared to stem cells, EVs present lower immunogenicity and tumorigenicity, cause fewer ethical problems, and have higher stability. Thus, EV therapy may have a broad clinical application in regenerative dentistry. Herein, we reviewed the currently available literature regarding the functional roles of EVs in oral and maxillofacial tissue regeneration, including in maxilla and mandible bone, periodontal tissues, temporomandibular joint cartilage, dental hard tissues, peripheral nerves and soft tissues. We also summarized the underlying mechanisms of actions of EVs and their delivery strategies for dental tissue regeneration. This review would provide helpful guidelines and valuable insights into the emerging potential of EVs in future research and clinical applications in regenerative dentistry.

## Introduction

The high prevalence of damage or loss in dental and maxillofacial tissue has garnered global attention, as it greatly impacts the quality of life of patients and imposes a substantial financial burden on society [1]. Exogenous transplantation or the use of maxillofacial prostheses are currently the most common treatments for repairing dental and maxillofacial tissue [2]. However, these therapies only serve to halt disease progression and are unable to fully restore the normal physiological structure and function [3]. As a result, there is a pressing need for new treatments that can achieve genuine regeneration of dental and maxillofacial tissues.

Regenerative medicine has emerged as a promising approach to replace repaired tissue to restore normal biological functions and reduce the reliance on transplantation [4]. In particular, mesenchymal stem cells (MSCs) have shown significant potential in this field by animal and clinical studies [5]. MSCs possess remarkable abilities for self-renewal, multilineage differentiation, and robust immunomodulation, making them pivotal players in tissue regeneration [6]. However, their use has been restricted in the clinic due to concerns regarding uncontrollability and potential transformation risks, underscoring the need for alternative cell-free therapies [7]. Recent findings have shed light on the fact that MSCs primarily exert their effects through the secretion of cytokines or membranous vesicles. These secreted substances regulate the microenvironment surrounding damaged tissues and orchestrate subsequent regeneration processes via paracrine signaling [8].

Extracellular vesicles (EVs) were recently revealed as the primary component of paracrine signals of cells [9]. They constitute a heterogeneous group of cell secretomes and are secreted by almost all cell types. Based on the size, EVs can be classified into three subtypes: microvesicles, exosomes, and apoptotic bodies [10]. The International Society for Extracellular Vesicles has collectively termed these subtypes as “EVs” [11]. Compared to cells, EVs are non-replicable and exhibit lower immunogenicity and improved biocompatibility [12]. They play a crucial role in promoting the proliferation and differentiation of targeted cells and regulating the entire process of tissue regeneration [13, 14]. Previous studies have demonstrated that EVs can aid their parent cells in performing physiological functions [15]. Subsequent investigations have further explored the functions and underlying mechanisms of EVs [16], and shown that EVs can activate specific signaling pathways to facilitate cellular communication through their unique contents, including proteins, nucleic acids, and signaling peptides [17].

Compared to other organs, the oral cavity has direct communication with the external environment, which provides a favorable condition for the implantation of EVs. This also avoids the issue of EVs traversing the circulatory system, thereby reducing any residual or cumulative effects in non-treated areas. Consequently, the application of exogenous EVs in dental regenerative medicine has been extensively studied and has shown promising treatment effects [18] . Therefore, EVs, with their non-mutating and non-duplicating characteristics, are considered promising tools for dental tissue regeneration [19].

**Fig. 1:**
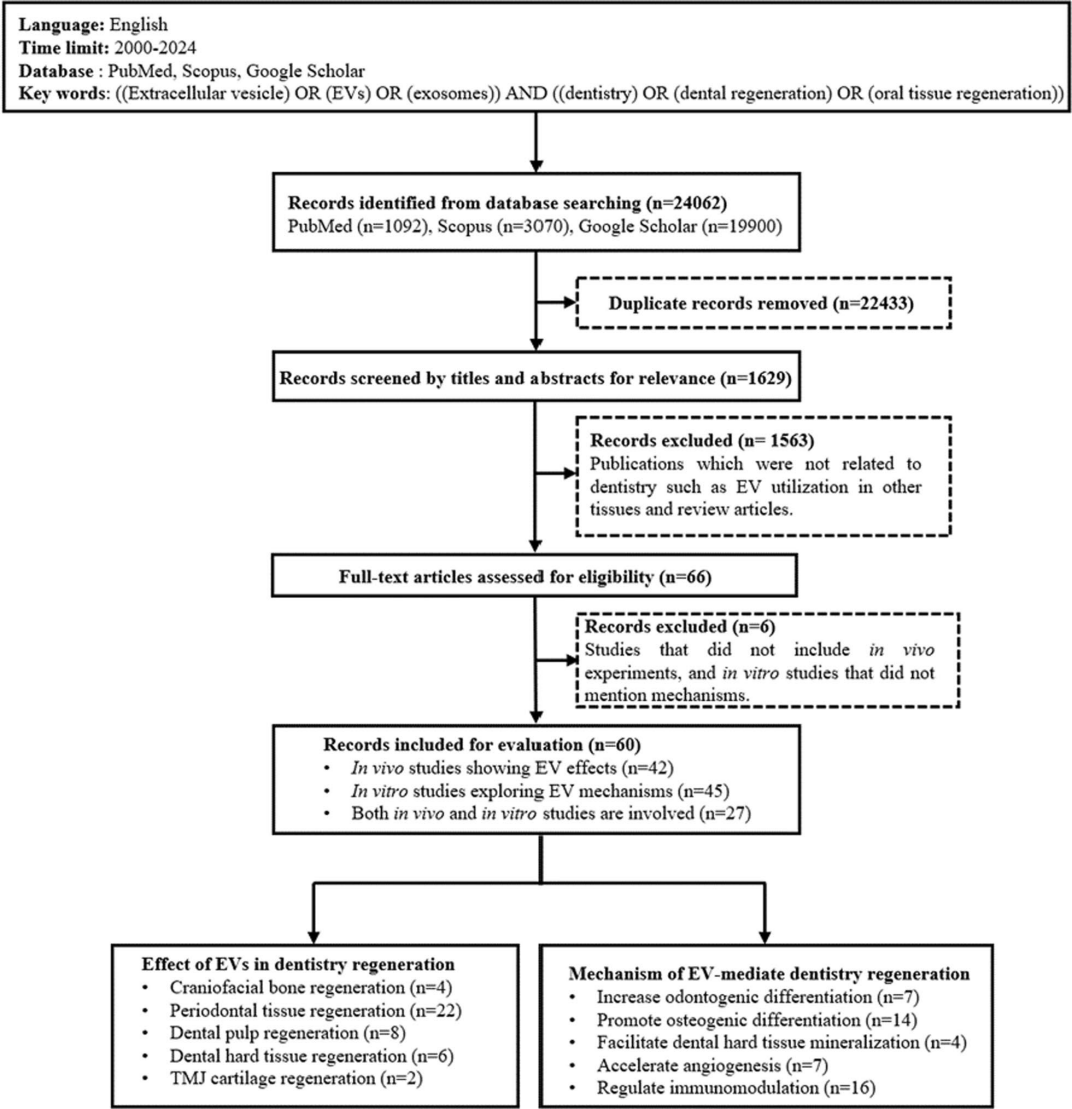
Flow chart of literature search for EVs in regenerative dentistry

**Fig. 2:**
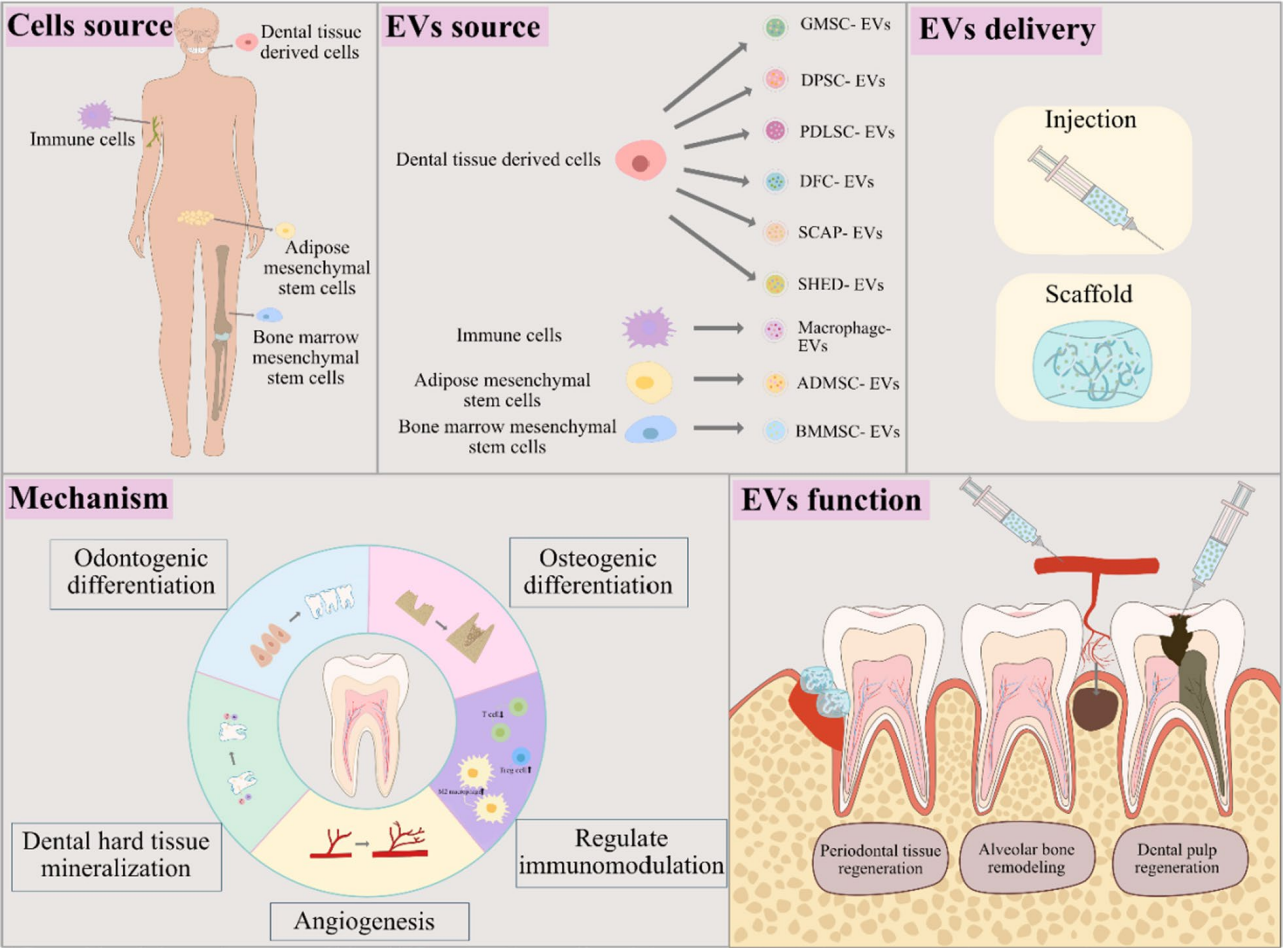
EVs parent cells source, EVs source, delivery strategies, functions and mechanisms of EVs in dental tissue regeneration. Abbreviations: EVs, extracellular vesicles; GMSC-EVs, gingival mesenchymal stem cell-derived extracellular vesicles; DPSC-EVs, dental pulp stem cell-derived extracellular vesicles; PDLSC-EVs, periodontal ligament stem cell-derived extracellular vesicles; DFC-EVs, dental follicle cell-derived extracellular vesicles; SCAP-EVs, stem cells from apical papilla-derived extracellular vesicles; SHED-EVs, human exfoliated deciduous teeth stem cell-derived extracellular vesicles; Macrophage-EVs, macrophage derived extracellular vesicles; ADMSC-EVs, adipose mesenchymal stem cell-derived extracellular vesicles; BMMSC-EVs, bone marrow mesenchymal stem cell-derived extracellular vesicles

In this review, we examine the current literature (Fig. 1), regarding the functional roles of EVs in oral and maxillofacial tissue regeneration, including their impact on maxilla and mandible bone, periodontal tissues, temporomandibular joint cartilage, dental hard tissues, peripheral nerves, and soft tissues. Additionally, we summarize the underlying mode of actions of EVs (Fig. 2) and discuss their delivery strategies in the applications of regenerative dentistry. The existing challenges and the prospect of the future for EVs in dentistry regeneration are also discussed. This literature search was conducted in three databases (PubMed, Scopus and Google Scholar). English publications were searched using the keywords ((extracellular vesicles) OR (EVs) OR (exosomes) AND (dentistry regeneration) OR (dental tissue regeneration) OR (oral tissue regeneration)). After reviewing the titles and abstracts, 60 selected publications with full texts were selected for detailed analysis. 3 research articles were duplicated in the part of mechanism of EV-mediate dentistry regeneration.

## Origin of EVs and their roles

EVs are nanoscale membrane vesicles and secreted by nearly all types of cells [20]. They are formed through the inward budding of multivesicular bodies that originate from late endosomal membrane invagination. These vesicles are subsequently released into the extracellular microenvironment by fusing with the plasma membrane [21]. During the process of EV formation, specific proteins, lipids, and nucleic acids are selectively recruited and encapsulated, granting EVs the ability to mediate paracrine crosstalk [22]. These proteins include adapter protein ALIX, endosome-related protein TSG101, and the transmembrane proteins CD9, CD63, and CD81 [23, 24]. The lipid bilayer of EVs typically comprises cholesterol, sphingomyelin, and phosphatidylserine, which significantly contribute to EV formation and their interaction with target cells [25, 26].

EVs interact with target cells through three main mechanisms, offering various avenues for studying signal pathways and therapeutic targets in different diseases [27]. Firstly, EVs engage in information transmission through receptor-ligand interactions, even without direct cell contact [15]. Secondly, EVs enhance cell adhesion properties by binding to the target cell membranes [28]. Lastly, EVs can fuse with the target cell membrane, delivering their contents into the cytoplasm and exerting biological effects [29]. The specific mechanisms of interaction depend on the composition and properties of EVs, as well as the characteristics of their parent cells [30]. Once released into the microenvironment, EVs transport their bioactive cargoes to specific cells, triggering a cascade of signaling pathways. The majority of EV components consist of proteins and nucleic acids, including DNA, mRNA, miRNA, tRNA, and non-coding RNA. While miRNA has been the focus of significant EV research due to its functional roles, more recent studies have indicated that proteins in EVs, rather than miRNA, play more critical roles in cell-cell communication.

## Dental tissue-derived EVs

**Fig. 3:**
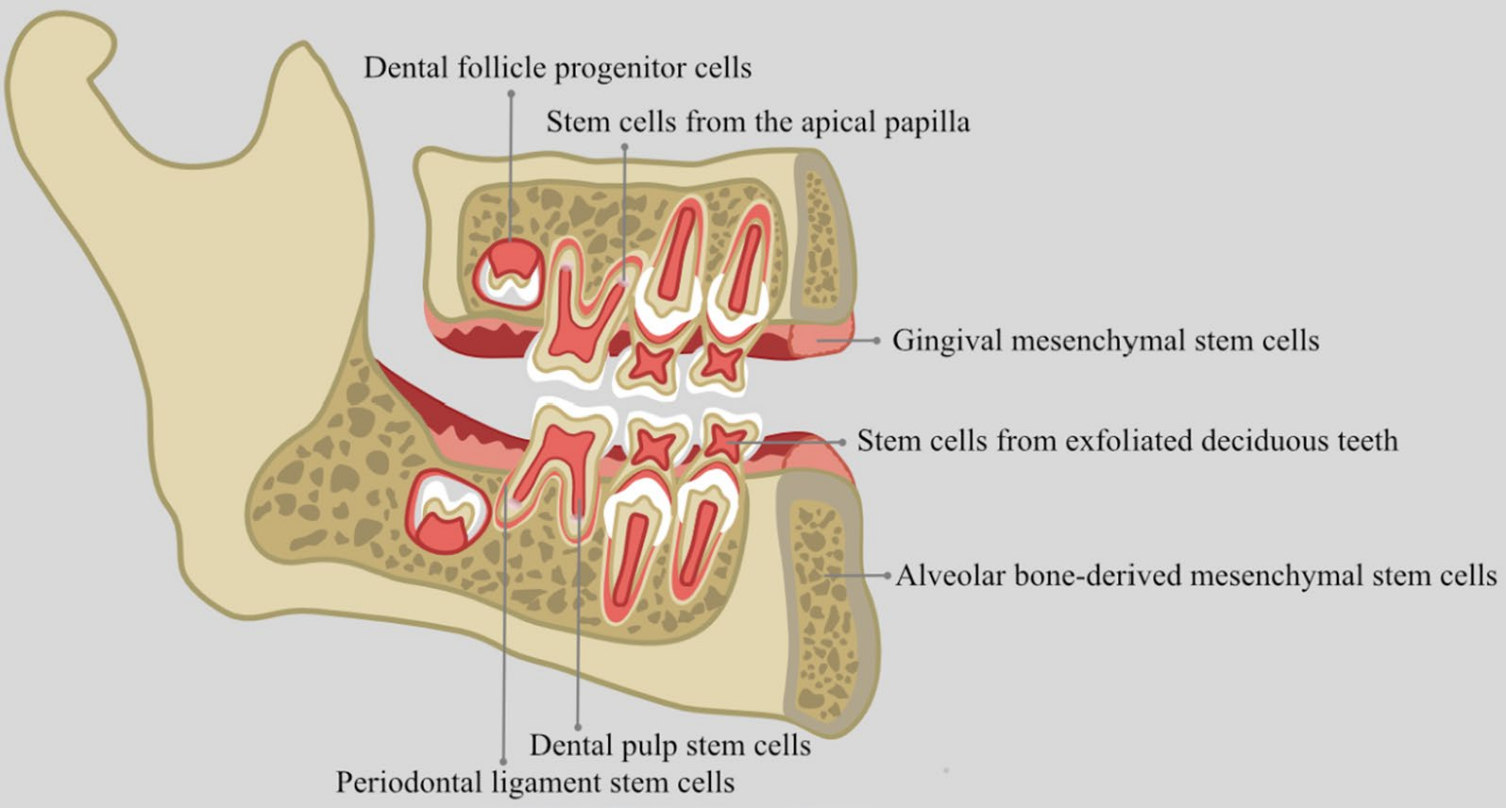
EVs from different dental tissue-derived stem cells used for dental tissue regeneration. EVs from different dental tissue-derived stem cells are mostly studied and utilized for dental tissue regeneration due to their multi-lineage differentiation and reproductive activity. EVs from different dental tissue-derived stem cells are mostly studied and utilized for dental tissue regeneration due to their multi-lineage differentiation and reproductive activity. Dental follicle progenitor cells are sourced from the connective tissue surrounding the developing tooth germ. Stem cells from the apical papilla are obtained from the apical papilla of incompletely developed teeth. Gingival mesenchymal stem cells are found within the gingiva. Stem cells from exfoliated deciduous teeth are harvested from the dental pulp of exfoliated primary teeth. Alveolar bone-derived mesenchymal stem cells can be extracted from the alveolar bone. Dental pulp stem cells are isolated from the dental pulp of permanent teeth. Periodontal ligament stem cells are sourced from the periodontal ligament of permanent teeth

The oral cavity constitutes a multifaceted environment encompassing diverse tissues, including jaws, periodontium, gingiva, teeth, oral mucosa, and glands. Saliva and gingival crevicular fluid create the fluid milieu within the oral cavity. In addition, various coatings envelop these tissues, each harboring an array of bacteria, collectively forming the bacterial biofilm [31]. All cells from these tissues and bacteria can secret EVs to participate in the dental tissue development. More importantly, these EVs shape the ecological environment of the oral cavity and oral environment in turn affect the stability and bioactivity of these EVs. However, although all EVs play certain roles in the dental tissue development, EVs from stem cells derived from different dental tissues are mostly studied and utilized for dental tissue regeneration due to their multi-lineage differentiation and reproductive activity. These stem cells include dental pulp stem cells (DPSCs), periodontal ligament stem cells (PDLSCs), dental follicle progenitor cells (DFCs), gingival mesenchymal stem cells (GMSCs), stem cells from the apical papilla (SCAPs), alveolar bone-derived mesenchymal stem cells (ABMSCs) and stem cells from exfoliated deciduous teeth (SHEDs) (Fig. 3). They were utilized in different oral tissue regeneration according to their specific regenerative characteristics.

Notably, EVs sourced from DPSCs (DPSC-EVs) have garnered significant attention in the field of dentistry regeneration owing to their remarkable osteo/odonto-inductive capabilities [32, 33]. Furthermore, DPSC-EVs have exhibited enhanced anti-necrotic, immunomodulatory, and anti-apoptotic properties compared to EVs derived from bone marrow mesenchymal stem cells (BMMSC-EVs) [34]. On the other hand, EVs originating from PDLSCs (PDLSC-EVs) have been demonstrated to upregulate the expression of CD31 and VEGFA to promote angiogenesis. Additionally, they fortify osteogenesis through the regulation of insulin, AMPK, and MAPK signaling pathways, while also modulating the Th17/Treg balance to bolster anti-inflammatory capabilities [35–38]. GMSC-EVs and ABMSC-EVs have also emerged as significant contributors to bone regeneration. They exhibit anti-osteoclastogenic activity and convey miR-1260 to inhibit inflammatory bone loss [39, 40]. Furthermore, when combined with a small intestinal submucosa-extracellular matrix, GMSC-EVs facilitate tongue lingual papillae repair and promote the recovery of taste buds [41]. Moreover, EVs derived from SCAPs (SCAP-EVs) hold great promise for dentistry regeneration. They enhance dentinogenesis of BMMSCs and are considered potential candidates for dentin-pulp regeneration [35]. Meanwhile, EVs sourced from SHEDs (SHED-EVs) effectively mitigate inflammation in temporomandibular joint diseases.

## Effect of EVs in regenerative dentistry

**Fig. 4:**
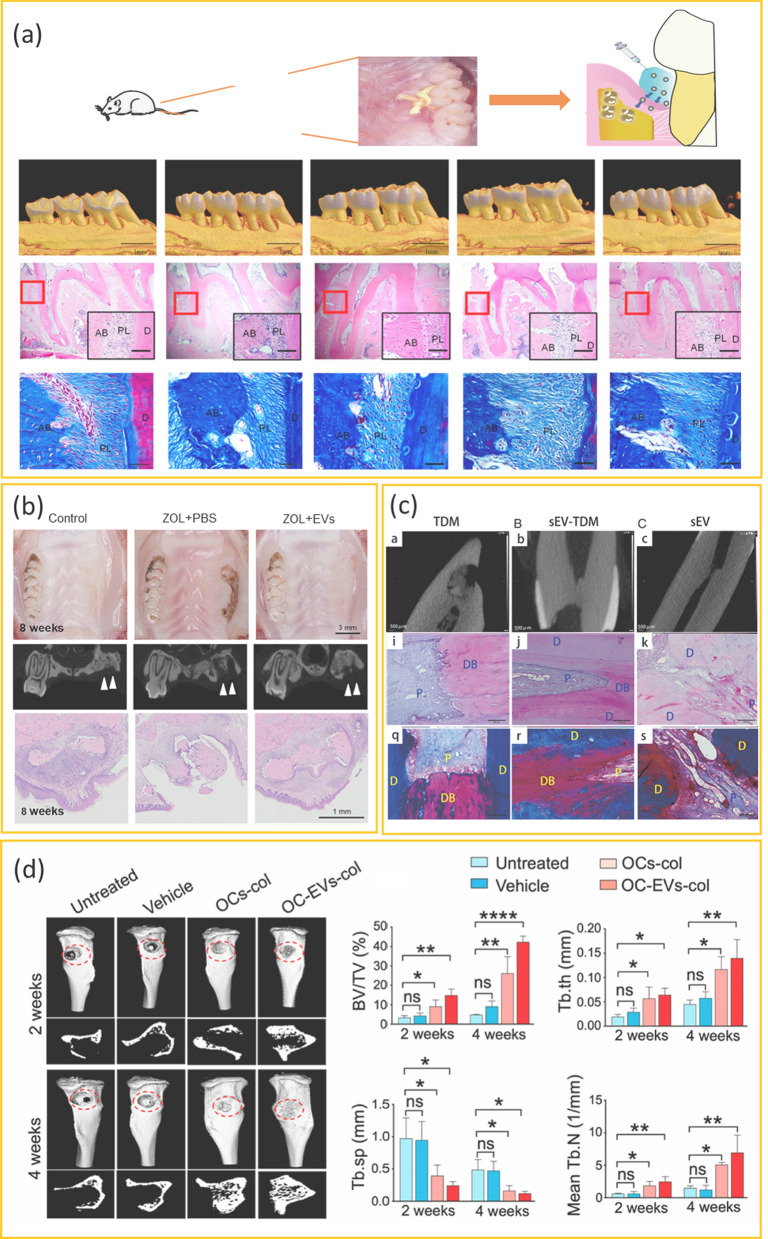
The therapeutic effects of extracellular vesicles on different dental tissue regeneration. **a** EVs derived from LPS-preconditioned DFCs loden on hydrogel applied in the treatment of periodontitis by repairing lost alveolar bone and promoting periodontal tissue regeneration. This figure is adapted and is freely accessible from reference [72], Licensed under a Creative Commons Attribution 4.0 International License (CC BY 4.0). **b** EVs derived from BMMSCs prevent BRONJ by preventing the spread of chronic inflammation and promoting angiogenesis and osteogenesis. This figure is adapted and is freely accessible from reference [46], **c** TDM and EVs isolated from DPSCs promote reparative dentin formation. This figure is adapted and is freely accessible from reference [87], Licensed by Sage Publications and Copyright Clearance Center. **d** EVs derived from osteoclasts promote bone regeneration. This figure is adapted and is freely accessible from reference [14], Reprinted under the terms of the Creative Commons CC-BY license. Abbreviations: AB, alveolar bone; PL, periodontal ligament; D, cementum; ZOL, zoledronic acid; TDM, dentin matrix; D, dentin; P, pulp tissue; DB, dental bridge; BV./TV., bone volume/total volume; OCs-col, osteoclasts on collagen; OC-EVs-col, EVs derived from osteoclasts on collagen

Compared to tissues such as liver, skin, and muscle, oral tissues are generally constantly exposed to microorganisms from food, drink, and the oral microbiome and have limited blood supply during the regeneration process. This greatly affect their ability to heal efficiently and makes them more vulnerable to infections and inflammation. Numerous studies have highlighted that EVs derived from various cells, particularly stem cells, exhibit beneficial effects such as pro-regenerative, pro-vascularization, anti-inflammatory, and anti-apoptotic properties, irrespective of their distinctiveness from different sources [42]. These minuscule vesicles have exhibited the capacity to regenerate bone, dental tissues, and cartilage, rendering them promising therapeutic agents in the field of dental tissue regeneration [43] (Table 1)(Fig. 4).

**Table 1:** Effect of different EVs on dental tissue regeneration

Regeneration tissue	EVs source	Delivery strategy	Study model	EVs concentration/dose	Outcome in vivo	References
Maxillofacial tissue	ADMSCs	Intravenous injection	Rat BRONJ model	1 µg/kg	After treatment with ADMSC-EVs, bone parameters, including BV/TV, Tb·N, Tb·Th were increased, whereas Tb·Sp declined. Moreover, fewer necrotic bones but more osteoclasts were found in the EVs group. There were also more collagen and vessels in the EVs group.	[45]
	BMMSCs	Intravenous injection	Rat BRONJ model	30 µg	In a rat BRONJ model, some genes in maxillary, such as p21 and pRB were upregulated. Moreover, osteoclasts in the EVs group were not apoptosis, which showed opposite results in the control group.	[46]
	BMMSCs	Injection for each limb	Rat proximal tibial condyles	19.2 µg	In a rabbit proximal tibial condyles implanting EVs model for three days, the bone density was higher by 47.2% than that in control groups on average.	[47]
	DPSCs	Collagen	Rat jaw-bone defect model	100 µg	In a rat mandibula defect model, rats treated with DPSC-EVs showed an increase in bone mineral density, and the thickness of the buccal-lingual increased as well.	[48]
	SCAPs	Injection locally	Mouse palatal gingiva wound healing model	40 µg	In the defect area of mice gingiva, after injecting SCAP-EVs, it formed newly epidermis and connective tissues, with high expressions of COL1 and fibronectin. Furthermore, there formed new blood vessels in the defect area.	[50]
	GMSCs	SIS-ECM laden with EVs	Rat critical-sized tongue defect model	Not mentioned	In a rat critical-sized tongue defect model, rat treated with SIS-ECM laden with GMSC-EVs facilitate recovery of epithelial layers and the regeneration of taste buds.	[41]
	EPI-NCSCs	Not mentioned	Rat FNI model	Not mentioned	The FNI rat recovered eyelid-buccal synkinesis, and the group treated with EPI-NCSCs showed higher amplitude during the blink reflex in the rats’ left buccal territory.	[49]
Periodontal tissue Dental-pulp	ADMSCs	Injection locally	Rat periodontitis model	80–150 µg	In a rat periodontitis model, rat injected ADMSC-EVs showed cementum, periodontal fibers and alveolar bone formation. Moreover, newly formed alveolar bone showed various reversal lines and new osteoid tissue.	[57]
	ADMSCs	PLGA/pDA	Rat subcutaneous transplantation with the tooth root slice model	Not mentioned	Periodontitis rats treated with ADMSC-EVs showed a few high-density spots and bone nodules on the edge of the defect area. Meanwhile, osteogenesis related genes such as ALP, RUNX2, OCN were upregulated.	[58]
	BMMSCs	Collagen	Rat periodontitis model	40 mg EVs/sponge	Periodontitis rats treated with BMMSC-EVs showed oriented periodontal fibers span between the newly-formed bone and root surface, and higher BV/TV was seen compared with the other groups.	[54]
	BMMSCs	Hydrogel	Rat periodontitis model	500 µg/ml	In a rat periodontitis model, rats treated with BMMSC-EVs on hydrogel had fewer TRAP positive cells with lower OPG/RANKL ratio, and fibers destruction and alveolar bone loss were lower as well.	[55]
	DPSCs	Chitosan hydrogel	Mouse periodontitis model	50 µg	Mice treated with DPSC-EVs showed a lower alveolar bone loss in experiment groups. Additionally, it showed the thicker epithelial tissue layers on periodontal bone.	[61]
	DPSCs	Injection locally	Mouse periodontitis model	1 million cells secretory EVs	In a mouse periodontitis model, injecting 3D-cultured EVs arose more alveolar bone and less inflammatory cells, which occurred less osteoclasts in the periodontium. In addition, proinflammatory genes including IL-1a, IL-1 β, Ccl12, TNF were downregulated.	[62]
	DPSCs	Injection locally	Mouse periodontitis model	75 million particles	In a mouse periodontitis model, EVs-treated group showed the least alveolar bone loss, while TRAP staining showed EVs inhibited osteoclast-like colonies.	[60]
	DPSCs	Gelatin	Mouse subcutaneous transplantation with the tooth root slice model	250 µl/scaffold	In contrast with the control group, more dentin and collagen were observed. Moreover, the account of DMP positive cells was higher.	[97]
	DFCs	Collagen	Rat periodontitis model	Not mentioned	New bone formation and newly bone showed denser outcomes compared to other groups. In addition, the higher thickness of trabecular was seen in the EVs-treatment group.	[64]
	DFCs (LPS-preconditioned)	Hydrogel	Rat periodontitis model	500 µg/ml	After treated DFC-EVs, the distance from the cementoenamel junction to the alveolar bone crest was shortened significantly, with obviously alveolar bone formation and periodontal tissue arranged in a dense.	[72]
	DFCs	Collagen	Mouse periodontitis model	40 µg	Rats treated with DFC-EVs had increased BV/TV, with more trabecular bone continuously and completely. Moreover, more staining for OCN, OPN were found and more osteogenesis-gene expressed (such as ALP, MMP-2).	[65]
	DFCs (LPS-preconditioned)	HA	Dog periodontitis model	200 µg	In a dog periodontitis model, HA carried DFC-EVs formed denser, more regular and wider periodontal ligaments, attaching new fibers to the cementum layer. Also significantly enhanced the bone parameters, including BV/TV and Tb·Sp.	[71]
	Dendritic cells	Injection locally/ intravenous injection	Mouse periodontitis model	200 million particles	Mice with periodontal diseases treated with EVs from dendritic cells by local injection promoted soft tissues covered on the alveolar bone better.	[59]
	GMSCs (TNF-α treated)	Injection locally	Mouse periodontitis model	200 µg	In a mouse periodontitis model, mice injected GMSC-EVs had less bone resorption and fewer TRAP-positive cells.	[39]
	GMSCs	PLGA	Rat periodontitis model	“full” EVs in PLGA	On the one hand, GMSC-EVs upregulated the expression of IL-10 and TGF-β significantly, whereas they downregulated the level of TNF-α. On the other hand, the defect area of rats’ alveolar bone was significantly increased. Furthermore, osteogenic makers such as BMP2, RUNX2 and OCN were highly expressed.	[66]
	M2-Macrophages	Injection locally	Mouse periodontitis model	30 µl	In a mouse periodontitis model, after injecting EVs derived from M2-macrophage, less bone loss was found compared with the no EVs group.	[56]
	PDLSCs	β-TCP	Rat periodontitis model	150 µg/µl	In a rat periodontitis model, β-TCP carried EVs to the defect area had positive functions, in which the surface of the alveolar bone was more smooth, the periodontal fiber was more regular, also more collagen formation. The gene expression levels of OCN and RUNX2 were upregulated.	[68]
	PDLSCs	Gel-Alg Hydrogel	Rat alveolar bone defect model	2 µg/µl	Rats treated with PDLSC-EVs formed more new bones. Similarly, the bone parameter of BV/TV in EVs groups was higher than that in no-treatment groups.	[67]
	SCAPs	Injection locally	Mouse periodontitis model	5 µg	Periodontitis mice injected with SCAP-EVs showed less bone resorption. In the periodontal tissue, the level of TNF-α and IL-8 also decreased.	[63]
	SHEDs	β-TCP	Rat periodontal defect model	2 µg/µl	Periodontal rats treated with β-TCP/EVs had gained more newly-bone and more collagen accompanied by new blood vessels than the control group. EVs also enhanced COL1, and increased BV/TV.	[69]
	Salivary	Injection locally	Mouse periodontitis model	Not mentioned	Exosomal miR-25-3p treatment group showed lower CD4^+^and CD8^+^T cells from TNF-α or IL-17, taking effect on developing diabetes-associated periodontitis.	[37]
	DPSCs	Collagen	Mouse tooth root slice model	1.25 million cells’ EVs in 1cm^2^ collagen membrane	In the root slice model, collagen membrane immobilized with DPSC-EVs increased odontogenic markers, such as DMP1, DPP on explant sections. The osteogenesis markers and angiogenesis markers such as vWF, BMP2, TGF-β, PDGF, RUNX2 were upregulated.	[76]
	DPSCs (LPS-preconditioned)	Peptide-hydrogel	Rat dental pulp removed model	200 µg/ml	In a pulp-removing rat model, treated by DPSC-EVs facilitated more blood vessels and newly connective tissue formation.	[79]
	DPSCs	1) Implantation; 2) Injection locally	(1) Mouse subcutaneous transplantation with the tooth root slice model; (2) Dog dental pulp removed model	Not mentioned	In the model of nude mice implanted tooth scaffold under dorsum, EV-treatment groups showed dentin-like tissue with higher vessel density; in the dog dental pulp removed model, after injecting EVs locally, it showed dental-pulp like tissue formation with high expression of DSPP and DMP-1.	[81]
	DPTs/DPCs	Collagen with SCAPs	Mouse in vivo implant model based on TDM	160 µg/ml	Pre-dentin-like tissue appeared with a number of blood vessels, and formed some odontoblast-like cells and dense fibers. Furthermore, more polarizing odontoblast-like cells were shown after DPT-EVs were treated.	[80]
	Hertwig’s epithelial root sheath cells	Collagen	Mouse subcutaneous transplantation with the tooth root slice model	2 mg/ml	It formed newly predentin-like tissue and odontoblast-like cells with collagen fibers, new blood and nerve nearby. Moreover, gene expressions of DSPP, DMP1 and β-catenin in the soft tissue around dentin also increased.	[78]
	Schwann cells	Collagen	Rat subcutaneous transplantation with the tooth root slice model	4 mg/ml	Mice treated with EVs from Schwann cells had significantly more blood vessels and pre-dentin-like tissues, and formed odontoblast-cells. Some proteins such as DSPP and DMP1 led to odontogenic differentiation were increased.	[77]
	SCAPs	Collagen	Mouse subcutaneous transplantation with the tooth root slice model	2 mg/ml	In a subcutaneous transplantation model with tooth root slice transplanted, EVs delivered by collagen formed newly regenerated tissues such as polarizing odontoblast-like cells, pre-dentin-like cells, collagen fibers with new blood vessels and nerves. Genes expression of DSPP, DMP1, β-catenin between dentin and soft fiber enhanced as well.	[35]
	SHEDs	Implantation locally	Mouse subcutaneous transplantation with the tooth root slice model	Not mentioned	After implanting SHED-EVs, there appeared continuous dentin layers with new blood vessels, which showed the regeneration of the dentin-pulp complex.	[75]
Dental hard tissues	Ameloblasts	–	–	–	EVs secreted from ameloblasts mediated biomineralization of enamel, showing more enamel crystals formation. Compared with the control group, the width of the enamel was increased, and the distance of the interrod was decreased.	[84]
	DPSCs (in odontogenic differentiation media)	PEG-PLGA-PEG	Rat Pulpotomy Model	1.25 mg/scaffold	In a rat-pulpotomy model, DPSC-EVs implanted into the pulp interface induced more complete dentin formation while showing special dentin-tubes and mineral tissues stained with abundant collagen.	[86]
	DPSCs	Implantation locally	Mini-pig pulp repair model	1 mg/µl	The group treated with EVs from DPSCs cooperated with treated dentin matrix showing the thickest mineralized layers of dentin, and appeared dentin-bridge. Moreover, osteoid dentin also be observed nearby the pulp tissue.	[87]
	M2-Macrophages	Intravenous injection	Mouse mechanical force induced OTM model	100 µg	In a mechanical force-induced orthodontic tooth movement model, in comparison with other phenotypes macrophages, treated with M2-macrophage EVs induced cementoblast mineralization formation, which showed highly-expressions of BSP, OCN and OSX.	[88]
	Incisor epithelium and mesenchyme cells	-	-	-	In a knocked-down Rab27a/b CD1 mouse tooth organ reconstitution model, which reduces the secretion of EVs from epithelial and mesenchyme cells, weaker dentinogenesis compared with control groups was seen.	[89]
TMJOA	EBCs	Injection locally	Rat TMJOA model	100 µg	In a TMJOA model, EBC-EVs displayed significant functions in bone parameters, including BV/TV, Tb·Th. The height, thickness and cellularity of cartilage were also significantly improved.	[92]

EVs, extracellular vesicles; ADMSCs, adipose mesenchymal stem cells; BMMSCs, bone marrow mesenchymal stem cells; DPSCs, dental pulp stem cells; DFCs, dental follicle cells; GMSCs, gingival mesenchymal stem cells; PDLSCs, periodontal ligament stem cells; SCAPs, stem cells from apical papilla; EPI-NCSCs, hair follicle epidermal neural crest stem cells; SHEDs, human exfoliated deciduous teeth stem cells; CM, serum-free conditioned medium; DPTs, dental pulp tissues; DPCs, dental pulp cells; EBCs, embryonic stem cell; LPS, lipopolysaccharide; SIS-ECM, small intestinal submucosa–extracellular matrix; PLGA, poly (lactic-co-glycolic acid); PLA, poly (lactic acid); HA, hyaluronic acid; β-TCP, β-tricalcium phosphate; Gel-Alg Hydrogel, gelatin-sodium alginate hydrogel; PEG-PLGA-PEG, polyethylene glycol-poly (lactic-co-glycolic acid)-polyethylene glycol; BRONJ, bisphosphonate related osteonecrosis of the jaw; TDM, treated dental matrix; FNI, facial nerve injury; OTM, orthodontic tooth movement; TMJOA, temporomandibular joint osteoarthrosis; BV/TV, bone volume/total volume; Tb·N, trabecular number; Tb·Th, trabecular thickness; Tb·Sp, trabecular separation; COL1, collagen 1; ALP, alkaline phosphatase; RUNX2, runt-related transcription factor 2; OCN, osteocalcin; TRAP, tartrate resistant acid phosphatase; OPG, osteoprotegerin; RANKL, receptor activator of nuclear factor-κB ligand; IL-1, interleukin-1; TNF, tumor necrosis factor; DMP-1, dentin matrix protein-1; OPN, osteopontin; MMP-2, matrix metalloproteinases-2; HA, hydroxyapatite; TGF, transforming growth factor; DPP, dentin phosphoprotein; vWF, vonWillebrandfactor; PDGF, platelet-derived growth factor; DSPP, dentin sialophosphoprotein; BSP, bone sialoprotein; OSX, osterix.

### Maxillofacial tissue regeneration

Maxillofacial diseases, such as congenital cleft palate, functional mandibular reconstruction, and conditions like odontogenic osteomyelitis or tumors, necessitate precise repair and functional restoration of the affected areas [44]. Noteworthy studies have demonstrated the efficacy of EVs in addressing these challenges [45]. For instance, in a model of bisphosphonate-related osteonecrosis of the jaw (BRONJ), the introduction of EVs derived from adipose mesenchymal stem cells (ADMSC-EVs) through tail vein injection in rats led to the formation of new jawbone and improvements in bone structure parameters [46]. BMMSC-EVs showcased preventive properties against the spread of chronic inflammation associated with aging cells. They further promoted osteogenesis and angiogenesis, effectively averting the occurrence of BRONJ [47]. DPSC-EVs implanted in a rat mandibular bone defect area also exhibited heightened jawbone density and facilitated the formation of new jawbone [48].

In addition to hard tissue regeneration, EVs have demonstrated promise in maxillofacial soft tissue regeneration. GMSC-EVs, when combined with small intestinal submucosa extracellular matrix, were implanted in a rat critical-sized tongue defect site, resulting in the regeneration of epithelial cells and the restoration of taste buds and lingual papilla [41]. Moreover, EVs derived from hair follicle epidermal neural crest stem cells, in conjunction with acellular nerve allografts, were employed to bridge facial nerve defects. This intervention led to thicker myelination and robust remyelination [49]. Additionally, SCAP-EVs enhanced angiogenesis and vascularization in a rat hard palate mucosa defect model [50].

### Periodontal regeneration

The periodontium, encompassing the gingiva, periodontal ligament, and alveolar bone, serves as the structural support for teeth [51]. Periodontitis, a widespread global issue, is characterized by the progressive deterioration of the periodontium and inflammation [52]. The ultimate objective of periodontal tissue regeneration is to foster the development of new periodontal bone, complete with fresh periodontal ligaments, and the reattachment of the gingiva [53].

Numerous studies have unveiled the remarkable regenerative potential of EVs in periodontal tissue regeneration. For example, BMMSC-EVs have demonstrated the ability to stimulate alveolar bone formation and repair periodontal ligaments in models of periodontitis [54, 55]. Moreover, EVs released by M2 macrophages have proven effective in preventing alveolar bone loss [56]. The injection of EVs from ADMSCs into rat periodontal pockets has resulted in the formation of cellular periodontal tissue perpendicular to the cementum and alveolar bone [57, 58]. Moreover, EVs derived from dendritic cells have exhibited potential in treating degenerative alveolar bone diseases by promoting the coverage of soft tissues over the alveolar bone [59].

Emerging evidence suggests that EVs derived from dental tissues also contribute significantly to periodontal tissue regeneration. For instance, DPSC-EVs and SCAP-EVs have been reported to inhibit alveolar bone loss [60–63]. EVs derived from dental follicle progenitor cells (DFC-EVs) have been shown to enhance the formation of denser alveolar bone with increased trabecular thickness compared to control groups [64, 65]. Gingival mesenchymal stem cell-derived EVs (GMSC-EVs), PDLSC-EVs and SHED-EVs have proven efficient in repairing alveolar bone defects, accompanied by the development of new blood vessels [66–69].

Given that periodontal diseases often involve inflammation, evaluating the function of EVs under inflammatory conditions is essential [70]. Research has demonstrated that EVs released from GMSCs treated with TNF-α effectively prevent periodontal bone resorption [39]. Furthermore, studies have shown that lipopolysaccharide (LPS)-preconditioned DFC-EVs promote the proliferation of PDLSCs and macrophages [71]. Similarly, LPS-preconditioned DFC-EVs have been found to be beneficial for the formation of integrated periodontal tissue in PDLSCs compared to healthy DFC-EVs [72].

### Dental pulp regeneration

The dental pulp, the sole soft tissue within a tooth, resides within the pulp cavity, encircled by dentin. It comprises connective tissue, blood vessels, and nerves, rendering it vascularized and innervated [73]. Consequently, endodontic regeneration is a multifaceted process encompassing not only dental pulp regeneration and dentin-pulp complex formation but also pulp revascularization and neurological recovery [74].

Numerous research studies have shed light on the role of EVs in fostering dentin-pulp regeneration. EVs derived from various sources, such as SHEDs, DPSCs, SCAPs, Hertwig’s epithelial root sheath cells, and Schwann cells, have been subcutaneously implanted into mice, resulting in the promotion of dentin-pulp regeneration [35, 75–78]. Rats treated with LPS-preconditioned DPSC-EVs exhibited the formation of dental pulp-like tissue replete with new blood vessels in a model where dental pulp had been removed [79]. In another study, collagen containing SCAPs were placed at the root tip, and the cavity was filled with EVs derived from dental pulp tissue/stem cells, leading to the regeneration of dense pulp-like tissue and predentin-like tissue [80]. Intriguingly, Li et al. reported that apoptotic bodies, typically regarded as indicators of cellular end-of-life, spurred the formation of dental pulp-like tissue replete with abundant blood vessels [81]. As mentioned earlier, neurological recovery is also vital for dental pulp regeneration, with research highlighting the potential effects of EVs on neuroregeneration, thereby underscoring the promise of EVs in pulp regeneration [82].

### Dental hard tissue regeneration and mineralization

Dental hard tissues encompass enamel, dentin, and cementum. Enamel is primarily composed of hydroxyapatite crystals, whereas dentin and cementum are a combination of hydroxyapatite and organic matrix [83]. Research has shown that EVs play a role in the formation and mineralization of dental hard tissues [84, 85].

In a rat pulpotomy model, DPSC-EVs prompted the creation of dentin tubes and reparative dentin bridges [86]. Another study illustrated how DPSC-EVs, in conjunction with dentin matrix, heightened the proliferation, migration, and odontogenesis of dental pulp cells, thus contributing to the continuous formation of reparative dentin [87]. Zhao et al. reported that EVs originating from macrophages with different polarization phenotypes had distinct effects on cementoblast mineralization. Specifically, EVs derived from M2 macrophages fostered cementum mineralization and curtailed root resorption [88]. Jiang et al. posited that EVs facilitated communication between epithelial and mesenchymal cells. Epithelial cell-derived EVs were found to stimulate mesenchymal cells to produce dentin sialoprotein (DSP) and partake in mineralization, while mesenchymal cell-derived EVs induced epithelial cells to generate ameloblastin and amelogenin [89]. Moreover, it was proposed that intracellular ameloblast secretory EVs played a role in enamel mineralization [84].

### Temporomandibular cartilage regeneration

The temporomandibular joint (TMJ) is an intricate joint, comprising the mandibular condyle and the articular surfaces of the temporal bone, both covered with dense articular cartilage [90]. Temporomandibular joint osteoarthrosis (TMJOA) is a degenerative disease characterized by an imbalance between the synthesis and degradation of the condylar matrix mediated by chondrocytes [91]. This imbalance leads to the breakdown of the condylar matrix, resulting in joint disorganization, biomechanical alterations, disruption of the microenvironmental homeostasis around cartilage cells, and inflammation [92]. Because the TMJ cavity is an enclosed joint space with well-defined boundaries, this enclosed space provides a contained environment for injected substances, preventing their immediate dispersion into the surrounding tissues. Therefore, it has great potential to employ therapeutic EVs for the treatment of TMJOA.

Shen et al. firstly demonstrated that extracellular vesicles derived from BMMSCs under hypoxic conditions can enhance the proliferation, migration, and anabolic capacity of chondrocytes in vitro. Moreover, they exhibited pro-chondrogenic potential in vivo [93]. Similarly, SHED-EVs have shown the capability to down-regulate the expression of proinflammatory factors and matrix metalloproteinases, indicating their potential to mitigate inflammation in the temporomandibular joint and prevent further cartilage damage [94]. Other than dental tissue derived EVs, EVs derived from human embryonic mesenchymal stem cells have been observed to enhance chondrogenesis, leading to the formation of new hyaline cartilage closely resembling healthy tissue in a rat model of TMJOA [95].

## Mechanisms of EV-mediate dentistry regeneration

**Fig. 5:**
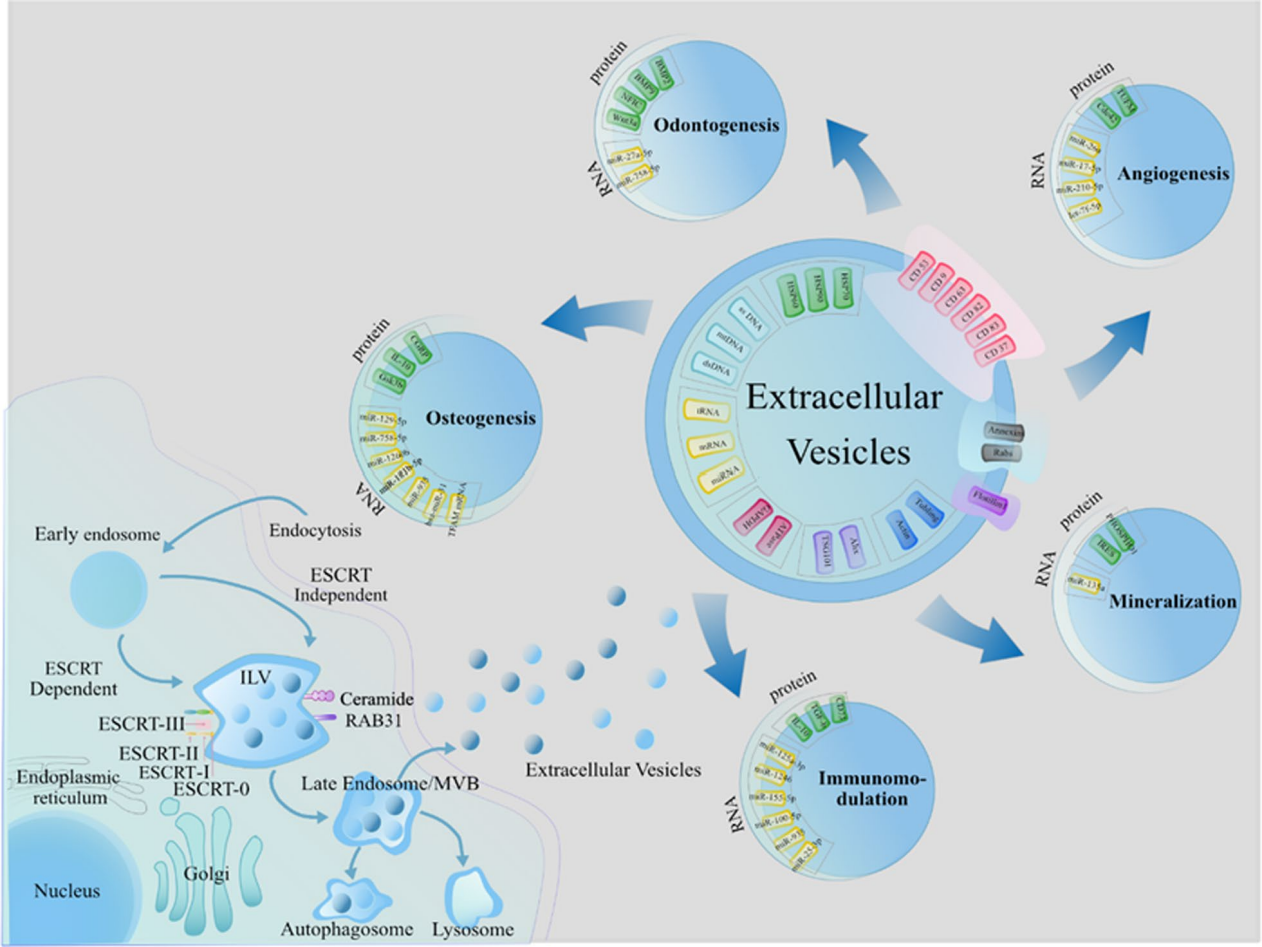
The effective components and functions of EVs for dental tissue regeneration. EVs are released upon the fusion of multivesicular bodies with plasma menbranes. They aid in dental tissue regeneration by promoting odontogenic differentiation, osteogenesis differentaition, dental hard tissue mineralization, angiogenesis and regulating immunomodultion through different cargos, including but not limited to protein, MicroRNA, and mRNA

**Fig. 6:**
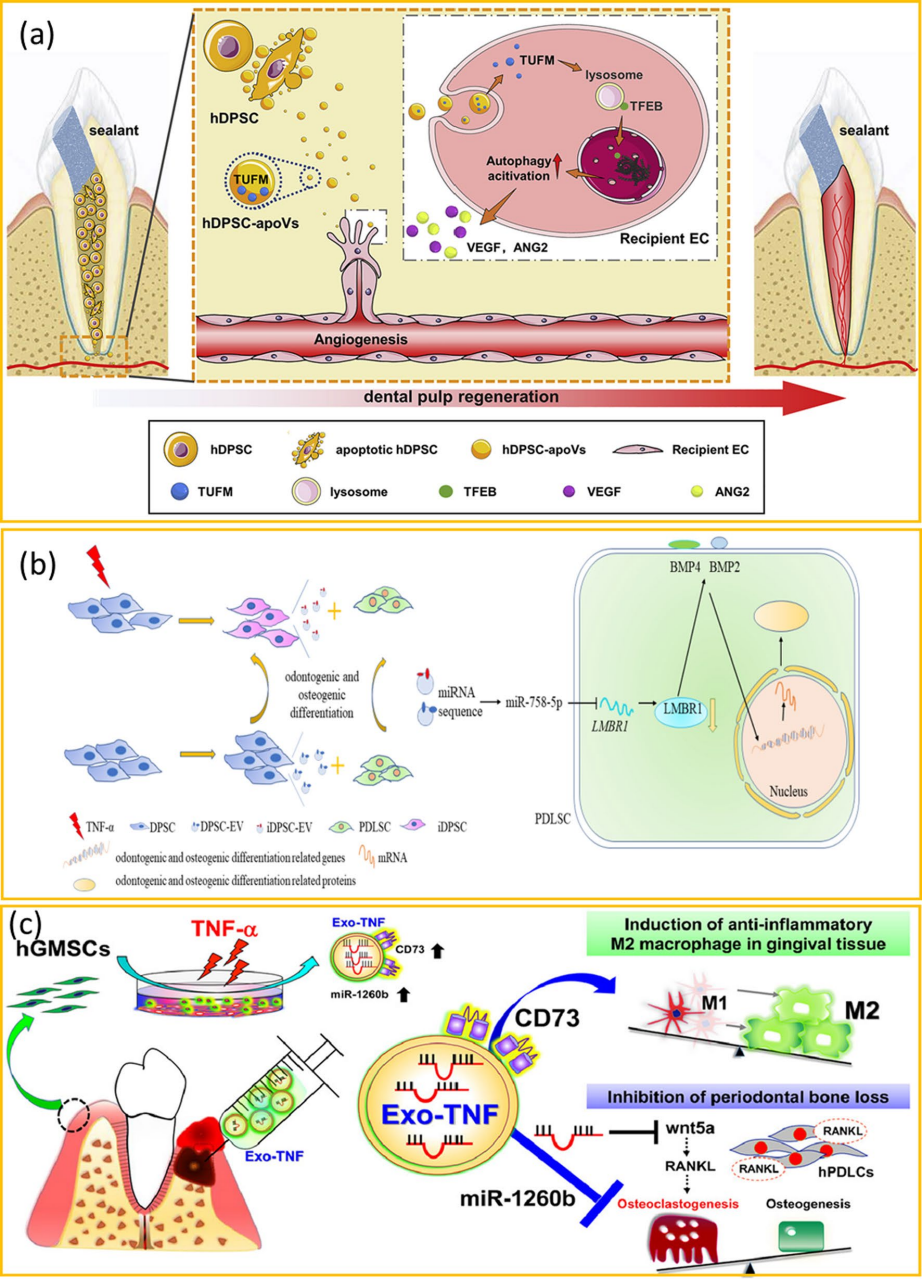
The mode of action of extracellular vesicles in promoting different dental tissue regeneration. **a** EVs derived from DPSCs specifically activate endogenous EC autophagy by transferring TUFM, thereby causing angiogenesis. The acceleration of vascular reconstruction promotes dental pulp regeneration. This figure is adapted and is freely accessible from reference [81], Licensed under a Creative Commons Attribution 4.0 International License (CC BY 4.0). **b** EVs derived from DPSCs under an inflammatory microenvironment participate in the regulating of odontogenic and osteogenic differentiation by miR-758-5p/LMBR1/BMP2/4 axis. This figure is adapted and is freely accessible from reference [99], Licensed under a Creative Commons Attribution 4.0 International License (CC BY 4.0). **c** EVs derived from GMSCs under inflammation microenvironment enhance M2-type macrophage polarization and prevent periodontal bone loss. This figure is adapted and is freely accessible from reference [39], Licensed under a Creative Commons Attribution 4.0 International License (CC BY 4.0). Abbreviations: EC, endothelial cells; hDPSC, human dental pulp stem cells; TUFM, Tu translation elongation factor, mitochondrial; TFEB, transcription factor EB; VEGF, vascular endothelial growth factor; ANG2, angiotensin 2; hDPSC-apoVs, apoptotic vesicles from human dental pulp stem cells; BMP, bone morphogenetic protein; LMBR1, limb development membrane protein 1; TNF-α, tumor necrosis factor α; DPSC-EV, EVs from dental pulp stem cells; iDPSC-EVs, EVs from dental pulp stem cells under inflammatory environment; PDLSC, periodontal ligament stem cells

Understanding these mechanisms is essential for harnessing the full regenerative potential of EVs. The mechanisms underlying extracellular vesicle-mediated dentistry regeneration are a complex and dynamic interplay of cellular and molecular processes. They facilitate key aspects of regeneration, including but not limited to osteogenesis, odontogenesis, mineralization of dental hard tissues, angiogenesis, immunomodulation (Table 2; Figs. 5 and 6). Through their ability to transfer these bioactive molecules, EVs modulate various signaling pathways, gene expression, and cellular behaviors, ultimately contributing to the repair and regeneration of dental tissues.

**Table 2:** Underlying mechanisms of EVs in the dental tissue regeneration

Functional effects	EVs source	EVs cargos	Involved signaling pathway	Outcome in vitro	References
Odontogenesis	DPSCs	BMP-2, BMP-9	P38 MAPK	DSPP	[76]
	DPSCs	miR-27a-5p	TGFβ-1/Smads	DSP↑, DMP-1 ↑	[98]
	DPSCs	NFIC	–	DSPP↑, DMP-1 ↑, ALP↑, NFSC ↑	[97]
	DPSCs (under inflammatory environment)	miR-758-5p	BMP	DSPP↑, DMP-1 ↑	[99]
	Hertwig’s epithelial root sheath cells	Wnt3a	Wnt/β-catenin	DSPP↑, DMP-1 ↑	[78]
Osteogenesis	ADMSCs	CGRP	-	RUNX2↑, ALP↑, OCN↑	[58]
	Plasma	miR-129-5p	FZD4/β-catenin	RUNX2↑, ALP↑, OPN↑	[104]
	DFCs	–	PLC/PKC/MAPK	ALP↑, OCN↑, OPN↑, MMP-2↑	[65]
	DFCs	–	P38 MAPK	RUNX2↑, ALP↑, BSP↑, COL1↑	[64]
	DPSCs (osteogenic-induced conditioned)	hsa-miR-31	–	RUNX2↑, COL1↑, OSX↑	[139]
	DPSCs (under inflammatory environment)	miR-758-5p	BMP	RUNX2↑, ALP↑, OCN↑	[99]
	GMSCs (TNF-α treated)	miR-1260b	NF-κB	RANKL/OPG↓	[39]
	M2-Macrophage	IL-10	IL-10/IL-10R	RUNX2↑, ALP↑, OCN↑, COL1a1↑	[56]
	Osteocyte	miR-181b-5p	PTEN/AKT	RUNX2↑, BMP-2↑, AKT1↑, P13KCA↑	[103]
	PDLSCs	Gsk3β	NF-κB	ALP↑, OCN↑, BMP-2↑	[101]
	PDLSCs	Gsk3β	Wnt	OCN↑, RUNX2↑	[68]
	SCAPs	miR-935	–	OCN↑, OPN↑	[63]
	SHEDs	-	AMPK	RUNX2↑, OPN↑, COL1↑	[69]
	SHEDs	TFAM mRNA	–	RUNX2↑, ALP↑, BMP-2↑	[102]
	UMSCs	–	P13k/AKT	RUNX2↑, ALP↑, OCN↑	[105]
Mineralization	17IIA11 cell lines	–	Erk1/2	Ca^2+^, Pi formation	[108]
	Ameloblast	PHOSPHO1	–	Enamel width↑, Interrod distance↓	[84]
	Incisor epithelial and mesenchymal cells	miR-135a	Wnt/β-catenin	COLIV↑, laminin↑, ALP↑, DSP↑, Bglap↑, Mineral nodule formation	[89]
	T4-4 cell lines	IRES	–	DSPP↑, DMP-1↑,DPP↑	[109]
Angiogenesis	DPSCs (from periodontally diseased teeth)	–	P38 MAPK	VEGF↑, MMP-9↑, KDR↑,	[114]
	DPSCs	TUFM	–	VEGF↑, ANG-2↑, MMP-9↑, HIF-1α↑	[81]
	PDLSCs	miR-17-5p	–	VEGFA↑	[111]
	SHEDs	–	AMPK	CD31↑, COL1↑	[69]
	SCAPs	Cdc42	–	CD31↑, vascular limen formation	[50]
	SHEDs	miR-26a	TGFβ/Smad2/3	VEGF↑, ANG-2↑, PDGF↑,	[75]
	SHEDs (hypoxic-preconditioned)	let-7f-5p, miR-210-5p	AGO/VEGF; miR-210-3p/ephrinA3	VEGF↑, MMP-9↑, ANGPT1↑	[113]
Immunomodulation	BMMSCs	–	OPG-RANKL-RANK	Arginase↑, CD163↑, TGF-β1↑, iNOS↓, CD86↓	[55]
	DFCs (LPS-preconditioned)	–	ROS/JNK	RANKL/OPG↓	[71]
	DPSCs	miR-125a-3p	TLR, NF-κB	IL-1ra↑, IL-10↑, IL-1β↓, IL-6↓, TNF-α↓	[118]
	DPSCs	miR-1246	NF-κB p65, p38 MAPK	CD206↑, Arginase↑, CD163↑, IL-1β↓, IL-6↓, TNF-α↓, iNOS↓, CD86↓	[61]
	DPSCs	miR-1246	–	Th17/Treg↓	[62]
	Dendritic cells	TGF-β, IL-10	–	Th17/Treg↓	[59]
	EBCs	CD73	AKT/ERK, AMPK	MMP13↓, NO↓, s-GAG↑	[92]
	GMSCs	–	NF-κB p65, Wnt5a	TNF-α↓, IL-1β↓, IL-10↑	[140]
	PDLSCs	–	NF-κB	IL-1β↓	[116]
	PDLSCs	miR-155-5p	–	Th17/Treg↓, RORC↓, STRT1↓, FOXP3↑	[38]
	SHEDs	miR-100-5p	–	IL-6↓, IL-8↓, MMP-1↓, MMP-3↓, MMP-9↓, MMP-13↓	[94]
	SCAPs	miR-935	–	IL-6↓, IL-8↓	[63]
	Salivary	miR-25-3p		IL-17↓	[37]

### EVs increase odontogenic differentiation

Odontogenic differentiation constitutes a pivotal process in tooth development, and emerging research affirms the role of extracellular vesicles in inducing odontogenic differentiation and upregulating the expression of dental-related markers such as dentin sialophosphoprotein (DSPP) and dental matrix protein (DMP) [35, 96]. Of note, DPSC-EVs have been observed to undergo cellular endocytosis in a dose-dependent manner, consequently activating the p38/MAPK pathway and intensifying odontogenic differentiation [76]. Moreover, DPSC-EVs have demonstrated their potential to transport nuclear factor I/C (NFIC), a pivotal transcription factor central to tooth development. This transport, in turn, promotes the proliferation, migration, and dentinogenesis of SCAPs [97]. Studies utilizing miRNA sequencing have unveiled alterations in miRNA profiles following the uptake of DPSC-EVs, underscoring the role of EVs in orchestrating odontogenic differentiation through the TGFβ1/Smads signaling pathway [98]. In addition, miR-758-5p transported by DPSC-EVs under inflammatory conditions has the capacity to stimulate BMP signaling to ultimately govern odontogenic differentiation [99]. Meanwhile, EVs originating from Hertwig’s Epithelial Root Sheath Cells have been shown to activate the Wnt/β-catenin pathway, thereby establishing a conducive microenvironment for odontogenic differentiation by fostering the connection between epithelial cells and mesenchymal cells [78].

### EVs promote osteogenic differentiation

Osteogenic differentiation plays a pivotal role in bone formation, including craniofacial and alveolar bone remodeling and repair [100]. Several studies have diligently explored the potential roles of EVs in osteogenesis. Reports indicate that PDLSC-EVs contributed to the alveolar bone regeneration by mitigating the overactivation of the Wnt signaling pathway and suppressing NF-κB activity of osteoprogenitor cells [68, 101]. Meanwhile, DFC-EVs have been shown to activate the MAPK pathway, aiding in the repair of alveolar bone defects [99, 100]. In contrast to DFC-EVs, SHED-EVs regulate osteogenesis through the AMPK pathway [69]. SHED-EVs transport mitochondrial transcription factor A mRNA, thereby instigating mitochondrial aerobic metabolism and consequently augmenting bone regeneration [102].

DPSC-EVs, GMSC-EVs, and SCAP-EVs are shown to promote the osteogenic differentiation of stem cells via the miRNAs they carry [39, 63, 99, 101]. For instance, miR-181b-5p found within osteocyte-derived EVs facilitates the osteogenic differentiation of PDLSCs through the PTEN/AKT pathway [103]. Conversely, miR-129-5p within plasma secretory EVs inhibits jawbone osteogenesis via the FZD4/β-catenin pathway [104]. Except for miRNA, mRNA and proteins in EVs can also contribute to dental bone regeneration by upregulating the osteogenic differentiations of stem cells. EVs derived from M2 macrophages transport IL-10 mRNA, activating the cellular IL-10/IL-10R pathway directly, thereby promoting osteogenesis and preserving bone homeostasis [56]. ADMSC-EVs expedite alveolar bone repair by transmitting calcitonin gene-related peptide (CGRP), a significant neuropeptide expressed during bone repair [58]. Meanwhile, umbilical cord mesenchymal stem cell-derived EVs (UMSC-EVs) were reported to enhance the osteoblastic differentiation capability of PDLSCs via the P13K/AKT pathway [105].

### EVs facilitate dental hard tissue mineralization

The mineralization process of dental hard tissue is a multifaceted phenomenon characterized by intricate interactions among various organic compounds [106]. Within this context, EVs serve as reservoirs of numerous factors that contribute to the formation of hydroxyapatite crystals and calcium phosphate [107]. Nevertheless, the precise mechanism through which EVs mediate mineralization remains the subject of debate and ongoing research. For instance, Chaudhary et al. demonstrated that EVs derived from the 17IIA11 cell line transport factors that induce enamel mineralization through the activation of the Erk1/2 pathway [108]. Another investigation proposed that miR-135a in EVs promote the reciprocal interaction between epithelial and mesenchymal cells, thereby activating the Wnt/β-catenin signaling pathway and facilitating the production of dentin matrix proteins [89]. Furthermore, researchers have postulated that ameloblast secretory EVs engage in interactions with orphan phosphatase 1 (PHOSPHO1) and play a role in amelogenesis [84]. Similarly, EVs have been found to participate in the transport of Dentin Phosphophoryn (DPP) to the extracellular matrix, thereby contributing to the mineralization process [109].

### EVs accelerate angiogenesis

Blood vessels play a pivotal role in delivering vital bioactive elements, encompassing growth factors, nutrients, and progenitor cells, to sites of regeneration, thereby contributing significantly to the maintenance of homeostasis [110]. Accumulating evidence suggests that EVs exhibit the capacity to expedite angiogenesis in the context of dental regeneration [111, 112]. This facilitation primarily hinges on the transfer of microRNA (miRNA) payloads encapsulated within EVs. For instance, PDLSC-EVs augmented the vascularization of dental periodontal ligaments through the transmission of vascular endothelial growth factor (VEGF) via miR-17-5p [111]. Similarly, SHED-EVs transferred miR-26a to initiate the TGF-β/Smad signaling pathway, thereby fostering angiogenesis [75]. Another investigation illustrated that SHED-EVs regulated angiogenesis via the activation of the AMPK signal pathway [69]. Moreover, SHED-EVs were reported to enhance angiogenesis even under hypoxic conditions, accomplished by the transfer of let-7f-5p and miR-210-3p, which respectively modulate the AGO1/VEGF and miR-210-3p/ephrinA3 signaling pathways [113].

In addition to miRNAs, proteins transported by EVs significantly contribute to the expediting of angiogenesis. SCAP-EVs mediated the action of Cdc42, thereby promoting vascularization and aiding in the repair of craniofacial soft tissue [50]. DPSC-EVs regulated the activation of angiogenesis by modulating the translation elongation factor Tu via the transcription factor EB (TFEB)-autophagy pathway [81]. Additionally, DPSC-EVs demonstrated the capacity to promote angiogenesis and activate the p38 MAPK pathway, showcasing substantial angiogenic potential for pulp regeneration [114].

### EVs regulate immunomodulation

Extracellular vesicles play a pivotal role in orchestrating immunoregulatory processes, with a significant contribution stemming from the encapsulated miRNAs. For instance, miR-100-5p within SHED-EVs [94], miR-935 in SCAP-EVs [63], and miR-25-3p found in salivary secretory EVs [37] have demonstrated the capacity to modulate immune responses. Notably, specific miRNAs encapsulated within EVs play immunomodulatory roles through different signaling pathways. For example, EVs derived from embryonic stem cells (EBC-EVs) have been shown to suppress inflammation through the activation of adenosine receptor-dependent AMPK and AKT/ERK signaling pathways [92]. The NF-κB transcription factor, known for its pivotal role in regulating inflammatory responses, is proved to be another key response element in the immunomodulation process of EVs [115]. Numerous studies have established that EVs exert influence over immune responses in the regenerative dentistry by modulating the NF-κB signaling pathway [61, 116–118]. In the realm of osteoimmunology, the RANKL (NF-κB ligand) and osteoprotegerin (OPG) system bear significance [71, 119]. EVs have demonstrated their ability to regulate the RANKL-RANK-OPG signaling within the context of osteoimmunology in dental bone regeneration [55]. Furthermore, the equilibrium between Th17 and Treg cells is revealed of importance in modulating inflammation to aid in the dental tissue regeneration [120]. EVs contribute significantly to this balance by virtue of various factors, including specific miRNAs like miR-1246 and miR-155-5p, as well as cytokines such as TGF-β and IL-10 [38, 59, 62].

## Delivery strategy of EVs

The delivery of extracellular vesicles represents a critical aspect of their utilization in various therapeutic contexts. Effective delivery approaches for EVs are essential to harness their regenerative and therapeutic potential. Various strategies have been developed to facilitate the precise and targeted delivery of EVs to specific tissues or cells, ranging from direct injection to more sophisticated engineered delivery systems. In the context of regenerative dentistry, delivering EVs to oral tissues has their own particularity due to the special oral anatomical and physiological characteristics. To be specific, the oral cavity is rich in saliva containing enzymes and chemicals and oral tissues are subject to constant mechanical stress due to activities such as chewing and speaking. All these factors might affect the stability and function of extracellular vesicles which have to be taken into consideration in the application EVs in regenerative dentistry.

### EVs delivery by injection

Due to the nano-size of EVs and well-established clinical procedure of intravenous injection, EVs have been initially and widely utilized through intravenous injection in various biomedical and therapeutic applications. In the field of regenerative dentistry, intravenous injection of EVs is proved to be feasible and effective. For example, in a BRONJ rat model, intravenously administered EVs were found to effectively modulate genes associated with osteogenesis and inflammation in the maxilla to promote bone regeneration [116]. However, EVs delivered systemically can become diluted in the bloodstream, which may reduce their concentration at the oral sites, potentially reducing the specificity of the treatment. Given the special oral anatomical structure, there is a preference for employing extracellular vesicles through direct injection into the target site in the context of regenerative dentistry regeneration. Research studies have provided evidence of the effectiveness of locally injected EVs in promoting the formation of new epidermal tissue and enhancing vascularization, as demonstrated in a mouse model of palatal gingiva wound healing [50]. Moreover, in an experimental model of alveolar bone loss, locally injected EVs exhibited slower clearance and demonstrated higher affinity compared to systemic injection [59]. However, it is crucial to acknowledge a significant limitation associated with the use of injected EVs, namely the stability and retention of EVs post-administration, particularly in the oral cavity. The special oral environment and the salivary flow can result in a notable loss of EVs, which significantly affect the therapeutic effectiveness and potential applications of injected EVs for the treatment of dental diseases.

### EVs delivery by carriers

The direct injection of EVs into target sites in the oral cavity presents challenges related to their stability and retention in vivo. In contrast, the utilization of carrier-based delivery systems, such as hydrogels and ceramics, offers notable advantages, primarily concerning controlled release and prolonged retention duration. This carrier-based EV delivery exhibits significant potential for augmenting the therapeutic effectiveness of EVs in the field of dental regenerative medicine [121, 122]. Given the superior biocompatibility and tunability, hydrogel materials are widely used to deliver cells and EVs via minimally invasive procedures for tissue regeneration. Collagen, a natural hydrogel derived from most tissues, stands out as a popular choice for delivering EVs in the realm of regenerative dentistry. When incorporated into collagen hydrogels, EVs have demonstrated their capacity to enhance osteogenesis, odontogenesis, and the regeneration of bone and dentin-like tissues across a spectrum of oral disease conditions [35, 48, 54, 55, 72]. Given that hydrogels have a texture similar to soft tissue, it is of great advantage that apply collagen hydrogels in the regeneration of dental pulp. Several studies have demonstrated that collagen hydrogels help promote angiogenesis, thus speeding up the rate of dental pulp regeneration [76–78, 80]. Gelatin, a collagen derivative, also exhibits controlled-release properties for EVs, amplifying their effectiveness in promoting dentin formation [97]. Furthermore, alternative hydrogels like chitosan and alginate hydrogel have found utility as carriers for EVs in dental regeneration endeavors [55, 61, 67, 101]. Additionally, synthetic polymers such as PLGA/pDA, PLGA, and PEG-PLGA-PEG, engineered with precision to control their physical and chemical attributes, offer a means of achieving more predictable and sustained EV release for dental tissue regeneration [58, 66, 86]. However, it’s worth noting that the sustained release of EVs primarily relies on the physical encapsulation provided by these hydrogels, which typically spans several days. Consequently, there is a growing interest in developing advanced materials capable of enabling long-term EV delivery in the context of regenerative dentistry, given the chronic nature of most oral diseases.

On the other hand, ceramics are frequently used to deliver EVs for hard tissue regeneration in oral diseases due to their mechanical properties such as hardness and corrosion resistance and chemical components that is calcium and phosphate ions. Hydroxyapatite (HA), which has been available on the market for clinical therapy since the 1970s, is considered the most conventional ceramics for regenerating dental hard tissues [123]. For instance, in an ectopic dentin regeneration model, the group utilizing EVs with HA exhibited significant formation of dentin-like tissue [71]. Another noteworthy ceramic material is beta-tricalcium phosphate (β-TCP) and it is known for its biodegradability. It promotes rapid bone tissue regeneration when used in conjunction with EVs in a periodontitis model. This structure facilitates angiogenesis and actively contributes to the formation of bone tissue [68, 69].

It is of note that a burst release frequently occurs when loading EVs in ceramic materials because EVs are mostly adsorbed on to the ceramic surfaces through hydrophilic action. The combination of exosome and hybrid scaffolds might exert better regenerative effects than organic or inorganic materials alone, which needs further investigations in the regenerative dentistry.

## Limitations and future perspectives

While extracellular vesicles have demonstrated significant progress in advancing dentistry regeneration [124–127], their widespread implementation in clinical trials is contingent upon addressing several limitations and challenges. First, EV composition exhibits dependency on various factors, including the cell type, donor age, state, and the microenvironment in which parent cells reside, all of which influence their functional roles [128]. For instance, the immune profiles of mesenchymal stem cell-derived EVs (MSC-EVs) have been substantiated age-dependent variations [129]. Furthermore, EVs derived from samples of differing ages exhibit disparate effects during the osteogenesis process and display varying degrees of efficacy in bone repair [130]. Consequently, it is imperative to investigate EVs from diverse contextual sources to unravel the underlying mechanisms that intricately govern their therapeutic efficacy.

The other challenge that hampers the potential utilization of extracellular vesicles for dentistry regeneration is a dependable method for isolating and purification of EVs from cells or bodily fluids. Current isolation methods encompass ultracentrifugation, size-exclusion chromatography, asymmetrical flow field-flow fractionation, and immunoaffinity-based techniques [131]. Comparative analyses of these methods have revealed variations in EV particle yield and purity [132]. For instance, one study compared the purification of serum-EVs using ultracentrifugation and Total Exosome Isolation reagent, with the latter displaying superior purity based on miRNA profiles [133]. Meanwhile, the conditions of the liquid for EV isolation, such as viscosity, preservation environment, and treatment methods, also influence EV purification outcomes. Nonetheless, a comprehensive comparison and optimization strategies for EV purification remains relatively unexplored. Moreover, these methods often incur high costs while yielding limited quantities of vesicles, thereby impeding their clinical applicability. Therefore, more advanced techniques for efficient and standardized EV isolation and purification are required for the future clinical application of EVs in regenerative dentistry. A relevant issue in this challenge is the standardization of EV usage, especially the determination of optimal dosages or concentrations. Notably, studies have reported a wide range of EV concentration/dosage across different investigations (Table 1). A general trend in the literature suggests that higher EV doses tend to yield relatively better tissue regeneration outcomes [134, 135], yet none of the studies provide definitive guidance regarding the optimal EV concentration for their respective animal models. To ensure consistency and efficacy, it is imperative to establish good manufacturing practices (isolation and purification) and comprehensive standards and guidelines for the clinical application of EVs [136].

The management of chronic dental diseases necessitates continuous engagement of extracellular vesicles. However, the sustained presence of EVs and their therapeutic effects at injury sites over extended periods remains a challenge. To address this, the development of a proficient delivery system for EVs offers distinct advantages in augmenting their therapeutic efficacy when integrated with modified scaffolds [137]. Consequently, forthcoming research endeavors may develop novel EV-loaded scaffolds, encompassing controlled release profiles, in vivo degradation characteristics, and loading efficiency. For instance, injectable microspheres with sustained release kinetics of EVs have been devised for addressing irregular tissue defects and for periodontitis [138]. More investigation on material-EV interaction would aid in optimizing the adaptability and plasticity of such scaffolds to ensure their effectiveness to deliver EVs.

## Conclusion

Extracellular vesicles have emerged as pivotal elements in cellular interactions and hold the potential to revolutionize regenerative dentistry by facilitating tissue regeneration, encompassing the maxillofacial, periodontal, dental, and temporomandibular cartilage regions. These vesicles, sourced from diverse origins, make substantial contributions to regenerative dentistry through various mechanisms, including the promotion of odontogenesis, osteogenesis, dental hard tissue mineralization, angiogenesis, and modulation of the immune response. Leveraging diverse delivery strategies has allowed for more effective utilization of EVs, enhancing their regenerative efficacy in the field of dental tissue regeneration. Nonetheless, the successful clinical translation of EV-based therapies hinges upon addressing several critical challenges. These include the optimization of EV yield, the establishment of a standardized definition for EVs, and the development of novel EV delivery strategies.

## Supplementary Information

Supplementary material 1

## Data Availability

Not applicable.

## Abbreviations

MSCs: Mesenchymal stem cells
EVs: Extracellular vesicles
ADMSCs: Adipose mesenchymal stem cells
ABMSCs: Alveolar bone-derived mesenchymal stem cells
BMMSCs: Bone marrow mesenchymal stem cells
DPSCs: Dental pulp stem cells
DFCs: Dental follicle progenitor cells
GMSCs: Gingival mesenchymal stem cells
PDLSCs: Periodontal ligament stem cells
SCAPs: Stem cells from the apical papilla
SHEDs: Stem cells from exfoliated deciduous teeth
UMSCs: Umbilical cord mesenchymal stem cells
ADMSC-EVs: Adipose mesenchymal stem cell-derived extracellular vesicles
ABMSC-EVs: Alveolar bone-derived mesenchymal stem cell-derived extracellular vesicles
BMMSC-EVs: Bone marrow mesenchymal stem cell-derived extracellular vesicles
DPSC-EVs: Dental pulp stem cell-derived extracellular vesicles
DFC-EVs: Dental follicle cell-derived extracellular vesicles
GMSC-EVs: Gingival mesenchymal stem cell-derived extracellular vesicles
PDLSC-EVs: Periodontal ligament stem cell-derived extracellular vesicles
SCAP-EVs: Stem cells from apical papilla-derived extracellular vesicles
SHED-EVs: Human exfoliated deciduous teeth stem cell-derived extracellular vesicles
Macrophage-EVs: Macrophage derived extracellular vesicles
EBC-EVs: Embryonic stem cells
MSC-EVs: Mesenchymal stem cell-derived EVs
LPS: Lipopolysaccharide
BRONJ: Bisphosphonate-related osteonecrosis of the jaw
TMJ: Temporomandibular joint
TMJOA: Temporomandibular joint osteoarthrosis
AB: Alveolar bone
PL: Periodontal ligament
ZOL: Zoledronic acid
TDM: Dentin matrix
DB: Dental bridge
DSPP: Dentin sialophosphoprotein
DMP: Dentin matrix protein
CGRP: Calcitonin gene-related peptide
PHOSPHO1: Orphan phosphatase 1
DPP: Dentin phosphophoryn
miRNA: MicroRNA
VEGF: Vascular endothelial growth factor
TFEB: Transcription factor EB
EC: Endothelial cells
OPG: Osteoprotegerin
HA: Hydroxyapatite
β-TCP: Beta-tricalcium phosphate
SIS-ECM: Small intestinal submucosa–extracellular matrix
PLGA: Poly (lactic-co-glycolic acid)
PLA: Poly (lactic acid)
Gel-Alg Hydrogel: Gelatin-sodium alginate hydrogel
PEG-PLGA-PEG: Polyethylene glycol-poly (lactic-co-glycolic acid)-polyethylene glycol
TDM: Treated dental matrix
FNI: Facial nerve injury
OTM: Orthodontic tooth movement
BV/TV: Bone volume/total volume
Tb·N: Trabecular number
Tb·Th: Trabecular thickness
Tb·Sp: Trabecular separation
COL1: Collagen 1
ALP: Alkaline phosphatase
RUNX2: Runt-related transcription factor 2
OCN: Osteocalcin
TRAP: Tartrate resistant acid phosphatase
RANKL: Receptor activator of nuclear factor-κB ligand
IL-1: Interleukin-1
TNF: Tumor necrosis factor
OPN: Osteopontin
MMP-2: Matrix metalloproteinases-2
TGF: Transforming growth factor
DPP: Dentin phosphoprotein
vWF: VonWillebrandfactor
PDGF: Platelet-derived growth factor
BSP: Bone sialoprotein
OSX: Osterix
BMP: Bone morphogenetic protein
NFIC: Nuclear factor I C
IRES: Internal ribosome entry site
TUFM: Tu translation elongation factor, mitochondrial
MAPK: Mitogen-activated protein kinase
FZD4: Frizzled 4 gene
AMPK: Adenosine5’-monophosphate (AMP)-activatedproteinkinase
AGO: Arginaute
RANK: Receptor activator of nuclear factor-κB
ROS: Reactive oxygen species
JNK: c-Jun N-terminal kinase
NFSC: Neuro-fuzzy signal classifier
OSX: Osterix
P13KCA: Phosphoinositide-3-kinase, catalytic, alpha gene
KDR: Kinase insert domain receptor
ANG-2: Angiopoietin-2
HIF-1: Hypoxia inducible factor-1
PDGF: Platelet-derived growth factor
ANGPT1: Angiopoietin-1 gene
TGF: Transforming growth factor
iNOS: Inducible nitric oxide synthase
IL: Interleukin
NO: Nitric oxide
s-GAG: Sulfated glycosaminoglycan
RORC: The nuclear receptor retinoic acid receptor-related orphan receptor-gamma
STRT1: Sodium ion trehalose transporter 1
FOXP3: Forkhead box P3

## References

[1] Peres MA, Macpherson LMD, Weyant RJ, Daly B, Venturelli R, Mathur MR, et al. Oral diseases: a global public health challenge. Lancet. 2019;394:249–60. 31327369 10.1016/S0140-6736(19)31146-8

[2] Fernandes G, Yang S. Application of platelet-rich plasma with stem cells in bone and periodontal tissue engineering. Bone Res. 2016;4:1–21. 10.1038/boneres.2016.36 PMC5153571 28018706

[3] Zhou X, Xu X, Li J, Hu D, Hu T, Yin W, et al. Oral health in China: from vision to action. Int J Oral Sci. 2018;10:1. 29343681 10.1038/s41368-017-0006-6 PMC5944598

[4] Uccelli A, Moretta L, Pistoia V. Mesenchymal stem cells in health and disease. Nat Rev Immunol. 2008;8:726–36. 19172693 10.1038/nri2395

[5] Qi K, Li N, Zhang Z, Melino G. Tissue regeneration: the crosstalk between mesenchymal stem cells and immune response. Cell Immunol. 2018;326:86–93. 29221689 10.1016/j.cellimm.2017.11.010

[6] Gao F, Chiu SM, Motan DAL, Zhang Z, Chen L, Ji H-L, et al. Mesenchymal stem cells and immunomodulation: current status and future prospects. Cell Death Dis. 2016;7:e2062–2062. 26794657 10.1038/cddis.2015.327 PMC4816164

[7] Gegout P-Y, Stutz C, Olson J, Batool F, Petit C, Tenenbaum H, et al., et al. Interests of exosomes in bone and periodontal regeneration: a systematic review. Cell Biol Transl Med 13 Stem Cells Dev Dis. 2020;13:67–87. 10.1007/5584_2020_593 33159304

[8] Toh WS, Foldager CB, Pei M, Hui JHP. Advances in mesenchymal stem cell-based strategies for cartilage repair and regeneration. Stem Cell Rev Rep. 2014;10:686–96. 24869958 10.1007/s12015-014-9526-z

[9] Kou M, Huang L, Yang J, Chiang Z, Chen S, Liu J, et al. Mesenchymal stem cell-derived extracellular vesicles for immunomodulation and regeneration: a next generation therapeutic tool? Cell Death Dis. 2022;13:580. 35787632 10.1038/s41419-022-05034-x PMC9252569

[10] Lui PPY. Mesenchymal stem cell-derived extracellular vesicles for the promotion of tendon repair - an update of literature. Stem Cell Rev Rep. 2021;17:379–89. 32785869 10.1007/s12015-020-10023-8

[11] Théry C, Witwer KW, Aikawa E, Alcaraz MJ, Anderson JD, Andriantsitohaina R, et al. Minimal information for studies of extracellular vesicles 2018 (MISEV2018): a position statement of the International Society for Extracellular Vesicles and update of the MISEV2014 guidelines. J Extracell Vesicles. 2018;7:1535750. 30637094 10.1080/20013078.2018.1535750 PMC6322352

[12] Fiore EJ, Domínguez LM, Bayo J, García MG, Mazzolini GD. Taking advantage of the potential of mesenchymal stromal cells in liver regeneration: cells and extracellular vesicles as therapeutic strategies. World J Gastroenterol. 2018;24:2427–40. 29930465 10.3748/wjg.v24.i23.2427 PMC6010941

[13] Bagno L, Hatzistergos KE, Balkan W, Hare JM. Mesenchymal stem cell-based therapy for cardiovascular disease: progress and challenges. Mol Ther. 2018;26:1610–23. 29807782 10.1016/j.ymthe.2018.05.009 PMC6037203

[14] Faqeer A, Wang M, Alam G, Padhiar AA, Zheng D, Luo Z, et al. Cleaved SPP1-rich extracellular vesicles from osteoclasts promote bone regeneration via TGFβ1/SMAD3 signaling. Biomaterials. 2023;303:122367. 38465579 10.1016/j.biomaterials.2023.122367

[15] Yáñez-Mó M, Siljander PR-M, Andreu Z, Bedina Zavec A, Borràs FE, Buzas EI, et al. Biological properties of extracellular vesicles and their physiological functions. J Extracell Vesicles. 2015;4:27066. 25979354 10.3402/jev.v4.27066 PMC4433489

[16] Tsiapalis D, O’Driscoll L. Mesenchymal stem cell derived extracellular vesicles for tissue engineering and regenerative medicine applications. Cells. 2020;9:991. 32316248 10.3390/cells9040991 PMC7226943

[17] Yuan F-L, Wu Q, Miao Z-N, Xu M-H, Xu R-S, Jiang D-L, et al. Osteoclast-derived extracellular vesicles: novel regulators of osteoclastogenesis and osteoclast–osteoblasts communication in bone remodeling. Front Physiol. 2018;9:628. 29910740 10.3389/fphys.2018.00628 PMC5992398

[18] Gholami L, Nooshabadi VT, Shahabi S, Jazayeri M, Tarzemany R, Afsartala Z, et al. Extracellular vesicles in bone and periodontal regeneration: current and potential therapeutic applications. Cell Biosci. 2021;11:1–21. 33436061 10.1186/s13578-020-00527-8 PMC7802187

[19] Andrukhov O, Behm C, Blufstein A, Rausch-Fan X. Immunomodulatory properties of dental tissue-derived mesenchymal stem cells: implication in disease and tissue regeneration. World J Stem Cells. 2019;11:604–17. 31616538 10.4252/wjsc.v11.i9.604 PMC6789188

[20] Cosenza S, Ruiz M, Maumus M, Jorgensen C, Noël D. Pathogenic or therapeutic extracellular vesicles in Rheumatic diseases: role of mesenchymal stem cell-derived vesicles. Int J Mol Sci. 2017;18:889. 28441721 10.3390/ijms18040889 PMC5412468

[21] Pishavar E, Luo H, Naserifar M, Hashemi M, Toosi S, Atala A, et al. Advanced hydrogels as exosome delivery systems for osteogenic differentiation of MSCs: application in bone regeneration. Int J Mol Sci. 2021;22:6203. 34201385 10.3390/ijms22126203 PMC8228022

[22] Denzer K, Kleijmeer MJ, Heijnen HF, Stoorvogel W, Geuze HJ. Exosome: from internal vesicle of the multivesicular body to intercellular signaling device. J Cell Sci. 2000;113:3365–74. 10984428 10.1242/jcs.113.19.3365

[23] Raposo G, Stoorvogel W. Extracellular vesicles: Exosomes, microvesicles, and friends. J Cell Biol. 2013;200:373–83. 23420871 10.1083/jcb.201211138 PMC3575529

[24] McBride JD, Rodriguez-Menocal L, Badiavas EV. Extracellular vesicles as biomarkers and therapeutics in dermatology: a focus on exosomes. J Invest Dermatol. 2017;137:1622–9. 28648952 10.1016/j.jid.2017.04.021

[25] Boilard E. Thematic Review series: exosomes and microvesicles: lipids as key components of their biogenesis and functions extracellular vesicles and their content in bioactive lipid mediators: more than a sack of microRNA. J Lipid Res. 2018;59:2037–46. 29678959 10.1194/jlr.R084640 PMC6210911

[26] Tan SS, Yin Y, Lee T, Lai RC, Yeo RWY, Zhang B, et al. Therapeutic MSC exosomes are derived from lipid raft microdomains in the plasma membrane. J Extracell Vesicles. 2013;2:22614. 10.3402/jev.v2i0.22614 PMC3873122 24371518

[27] Racchetti G, Meldolesi J. Four distinct cytoplasmic structures generate and release specific vesicles, thus opening the way to intercellular communication. Extracell Vesicles Criculating Nucleic Acids. 2023;4:44–58. 10.20517/evcna.2023.03 PMC11648438 39698300

[28] Maas SLN, Breakefield XO, Weaver AM. Extracellular vesicles: unique intercellular delivery vehicles. Trends Cell Biol. 2017;27:172–88. 27979573 10.1016/j.tcb.2016.11.003 PMC5318253

[29] Mulcahy LA, Pink RC, Carter DRF. Routes and mechanisms of extracellular vesicle uptake. J Extracell Vesicles. 2014;3:24641. 10.3402/jev.v3.24641 PMC4122821 25143819

[30] Midekessa G, Godakumara K, Ord J, Viil J, Lättekivi F, Dissanayake K, et al. Zeta potential of extracellular vesicles: toward understanding the attributes that determine colloidal stability. ACS Omega. 2020;5:16701–10. 32685837 10.1021/acsomega.0c01582 PMC7364712

[31] Aas JA, Paster BJ, Stokes LN, Olsen I, Dewhirst FE. Defining the normal bacterial flora of the oral cavity. J Clin Microbiol. 2005;43:5721–32. 16272510 10.1128/JCM.43.11.5721-5732.2005 PMC1287824

[32] Mai Z, Chen H, Ye Y, Hu Z, Sun W, Cui L, et al. Translational and clinical applications of Dental Stem Cell-Derived exosomes. Front Genet. 2021;12:750990. 34764982 10.3389/fgene.2021.750990 PMC8576041

[33] Imanishi Y, Hata M, Matsukawa R, Aoyagi A, Omi M, Mizutani M, et al. Efficacy of extracellular vesicles from dental pulp stem cells for bone regeneration in rat calvarial bone defects. Inflamm Regen. 2021;41:1–10. 33853679 10.1186/s41232-021-00163-w PMC8048358

[34] Ji L, Bao L, Gu Z, Zhou Q, Liang Y, Zheng Y, et al. Comparison of immunomodulatory properties of exosomes derived from bone marrow mesenchymal stem cells and dental pulp stem cells. Immunol Res. 2019;67:432–42. 31407157 10.1007/s12026-019-09088-6

[35] Zhuang X, Ji L, Jiang H, Liu Y, Liu X, Bi J et al. Exosomes Derived from Stem cells from the apical papilla promote dentine-pulp complex regeneration by inducing specific dentinogenesis. Stem Cells Int. 2020;1:1–10. 10.1155/2020/5816723 PMC7273441 32565828

[36] Liu T, Hu W, Zou X, Xu J, He S, Chang L et al. Human periodontal ligament stem cell-derived exosomes promote bone regeneration by altering MicroRNA profiles. Stem Cells Int. 2020;1:1–13. 10.1155/2020/8852307 PMC7691010 33293963

[37] Byun J-S, Lee HY, Tian J, Moon JS, Choi J, Lee S-H, et al. Effect of salivary exosomal mir-25-3p on Periodontitis with insulin resistance. Front Immunol. 2022;12:775046. 35069547 10.3389/fimmu.2021.775046 PMC8777127

[38] Zheng Y, Dong C, Yang J, Jin Y, Zheng W, Zhou Q, et al. Exosomal microRNA-155‐5p from PDLSCs regulated Th17/Treg balance by targeting sirtuin‐1 in chronic periodontitis. J Cell Physiol. 2019;234:20662–74. 31016751 10.1002/jcp.28671

[39] Nakao Y, Fukuda T, Zhang Q, Sanui T, Shinjo T, Kou X, et al. Exosomes from TNF-α-treated human gingiva-derived MSCs enhance M2 macrophage polarization and inhibit periodontal bone loss. Acta Biomater. 2021;122:306–24. 33359765 10.1016/j.actbio.2020.12.046 PMC7897289

[40] Han P, Johnson N, Abdal-hay A, Moran CS, Salomon C, Ivanovski S. Effects of periodontal cells‐derived extracellular vesicles on mesenchymal stromal cell function. J Periodontal Res. 2023;58:1188–200. 37605485 10.1111/jre.13171

[41] Zhang Y, Shi S, Xu Q, Zhang Q, Shanti RM, Le AD. SIS-ECM laden with GMSC-derived exosomes promote taste bud regeneration. J Dent Res. 2019;98:225–33. 30335555 10.1177/0022034518804531

[42] Li M, Fang F, Sun M, Zhang Y, Hu M, Zhang J. Extracellular vesicles as bioactive nanotherapeutics: an emerging paradigm for regenerative medicine. Theranostics. 2022;12:4879–903. 35836815 10.7150/thno.72812 PMC9274746

[43] Vafaei S, Mansoori M, Hashemi F, Basiri M. Exosome odyssey to original line in dental regeneration. J Oral Biosci. 2022;64:271–8. 35589074 10.1016/j.job.2022.05.002

[44] Zhang Z. Bone regeneration by stem cell and tissue engineering in oral and maxillofacial region. Front Med. 2011;5:401–13. 22198752 10.1007/s11684-011-0161-7

[45] Huang J, Xiong J, Yang L, Zhang J, Sun S, Liang Y. Cell-free exosome-laden scaffolds for tissue repair. Nanoscale. 2021;13:8740–50. 33969373 10.1039/d1nr01314a

[46] Watanabe J, Sakai K, Urata Y, Toyama N, Nakamichi E, Hibi H. Extracellular vesicles of stem cells to Prevent BRONJ. J Dent Res. 2020;99:552–60. 32119600 10.1177/0022034520906793

[47] Maiborodin I, Shevela A, Matveeva V, Morozov V, Toder M, Krasil’nikov S, et al. First experimental study of the influence of Extracellular vesicles derived from multipotent stromal cells on Osseointegration of Dental implants. Int J Mol Sci. 2021;22:8774. 34445482 10.3390/ijms22168774 PMC8395855

[48] Lee AE, Choi JG, Shi SH, He P, Zhang QZ, Le AD. DPSC-derived extracellular vesicles promote rat Jawbone regeneration. J Dent Res. 2022;102:313–21. 36348514 10.1177/00220345221133716

[49] Pan Y, Tang L, Dong S, Xu M, Li Q, Zhu G. Exosomes from hair follicle epidermal neural crest stem cells promote acellular nerve allografts to bridge rat facial nerve defects. Stem Cells Dev. 2023;32:1–11. 36453239 10.1089/scd.2022.0245

[50] Liu Y, Zhuang X, Yu S, Yang N, Zeng J, Liu X, et al. Exosomes derived from stem cells from apical papilla promote craniofacial soft tissue regeneration by enhancing Cdc42-mediated vascularization. Stem Cell Res Ther. 2021;12:76. 33482924 10.1186/s13287-021-02151-w PMC7821694

[51] Nanci A, Bosshardt DD. Structure of periodontal tissues in health and disease*. Periodontol. 2000. 2006;40:11–28. 16398683 10.1111/j.1600-0757.2005.00141.x

[52] Joshipura V, Yadalam U, Brahmavar B. Aggressive periodontitis: a review. J Int Clin Dent Res Organ. 2015;7:11.

[53] Deas DE, Mealey BL. Response of chronic and aggressive periodontitis to treatment. Periodontol. 2000. 2010;53:154–66. 20403111 10.1111/j.1600-0757.2009.00334.x

[54] Chew JRJ, Chuah SJ, Teo KYW, Zhang S, Lai RC, Fu JH, et al. Mesenchymal stem cell exosomes enhance periodontal ligament cell functions and promote periodontal regeneration. Acta Biomater. 2019;89:252–64. 30878447 10.1016/j.actbio.2019.03.021

[55] Liu L, Guo S, Shi W, Liu Q, Huo F, Wu Y, et al. Bone marrow mesenchymal stem cell-derived small extracellular vesicles promote periodontal regeneration. Tissue Eng Part A. 2021;27:962–76. 32962564 10.1089/ten.TEA.2020.0141

[56] Chen X, Wan Z, Yang L, Song S, Fu Z, Tang K, et al. Exosomes derived from reparative M2-like macrophages prevent bone loss in murine periodontitis models via IL-10 mRNA. J Nanobiotechnol. 2022;20:110. 10.1186/s12951-022-01314-y PMC8898524 35248085

[57] Mohammed E, Khalil E, Sabry D. Effect of adipose-derived stem cells and their exo as adjunctive therapy to nonsurgical Periodontal treatment: a histologic and histomorphometric study in rats. Biomolecules. 2018;8:167. 30544734 10.3390/biom8040167 PMC6316309

[58] Yang Y, Zhang B, Yang Y, Peng B, Ye R. PLGA Containing human adipose-derived stem cell-derived extracellular vesicles accelerates the repair of alveolar bone defects via transfer of CGRP. Oxid Med Cell Longev. 2022;1:1–14. 10.1155/2022/4815284 PMC9206573 35726333

[59] Elashiry M, Elashiry MM, Elsayed R, Rajendran M, Auersvald C, Zeitoun R, et al. Dendritic cell derived exosomes loaded with immunoregulatory cargo reprogram local immune responses and inhibit degenerative bone disease *in vivo*. J Extracell Vesicles. 2020;9:1795362. 32944183 10.1080/20013078.2020.1795362 PMC7480413

[60] Shimizu Y, Takeda-Kawaguchi T, Kuroda I, Hotta Y, Kawasaki H, Hariyama T, et al. Exosomes from dental pulp cells attenuate bone loss in mouse experimental periodontitis. J Periodontal Res. 2022;57:162–72. 34826339 10.1111/jre.12949

[61] Shen Z, Kuang S, Zhang Y, Yang M, Qin W, Shi X, et al. Chitosan hydrogel incorporated with dental pulp stem cell-derived exosomes alleviates periodontitis in mice via a macrophage-dependent mechanism. Bioact Mater. 2020;5:1113–26. 32743122 10.1016/j.bioactmat.2020.07.002 PMC7371600

[62] Zhang Y, Chen J, Fu H, Kuang S, He F, Zhang M, et al. Exosomes derived from 3D-cultured MSCs improve therapeutic effects in periodontitis and experimental colitis and restore the Th17 cell/Treg balance in inflamed periodontium. Int J Oral Sci. 2021;13:43. 34907166 10.1038/s41368-021-00150-4 PMC8671433

[63] Zhang T, Chen Z, Zhu M, Jing X, Xu X, Yuan X et al. Extracellular vesicles derived from human dental mesenchymal stem cells stimulated with low-intensity pulsed ultrasound alleviate inflammation-induced bone loss in a mouse model of periodontitis. Genes Dis. 2022;4:1613–25. 10.1016/j.gendis.2022.06.009 PMC10311020 37397561

[64] Ma L, Rao N, Jiang H, Dai Y, Yang S, Yang H, et al. Small extracellular vesicles from dental follicle stem cells provide biochemical cues for periodontal tissue regeneration. Stem Cell Res Ther. 2022;13:1–18. 35241181 10.1186/s13287-022-02767-6 PMC8895915

[65] Yi G, Zhang S, Ma Y, Yang X, Huo F, Chen Y, et al. Matrix vesicles from dental follicle cells improve alveolar bone regeneration via activation of the PLC/PKC/MAPK pathway. Stem Cell Res Ther. 2022;13:1–20. 35093186 10.1186/s13287-022-02721-6 PMC8800263

[66] Zarubova J, Hasani-Sadrabadi MM, Dashtimoghadam E, Zhang X, Ansari S, Li S, et al. Engineered delivery of dental stem‐cell‐derived extracellular vesicles for periodontal tissue regeneration. Adv Healthc Mater. 2022;11:2102593. 10.1002/adhm.202102593 PMC9233004 35191610

[67] Zhao Y, Gong Y, Liu X, He J, Zheng B, Liu Y. The experimental study of periodontal ligament stem cells derived exosomes with hydrogel accelerating bone regeneration on alveolar bone defect. Pharmaceutics. 2022;14:2189. 36297624 10.3390/pharmaceutics14102189 PMC9611133

[68] Lei F, Li M, Lin T, Zhou H, Wang F, Su X. Treatment of inflammatory bone loss in periodontitis by stem cell-derived exosomes. Acta Biomater. 2022;141:333–43. 34979326 10.1016/j.actbio.2021.12.035

[69] Wu J, Chen L, Wang R, Song Z, Shen Z, Zhao Y, et al. Exosomes secreted by stem cells from human exfoliated deciduous Teeth promote alveolar bone defect repair through the regulation of Angiogenesis and Osteogenesis. ACS Biomater Sci Eng. 2019;5:3561–71. 33405738 10.1021/acsbiomaterials.9b00607

[70] Liu C, Seneviratne CJ, Palma C, Rice G, Salomon C, Khanabdali R, et al. Immunoaffinity-enriched salivary small extracellular vesicles in periodontitis. Extracell Vesicles Criculating Nucleic Acids. 2023;4:698–712. 10.20517/evcna.2023.48 PMC11648426 39697803

[71] Huang Y, Liu Q, Liu L, Huo F, Guo S, Tian W. Lipopolysaccharide-preconditioned dental follicle stem cells derived small extracellular vesicles treating periodontitis via reactive oxygen species/Mitogen-activated protein kinase signaling-mediated antioxidant effect. Int J Nanomed. 2022;17:799–819. 10.2147/IJN.S350869 PMC8882029 35228798

[72] Shi W, Guo S, Liu L, Liu Q, Huo F, Ding Y, et al. Small extracellular vesicles from lipopolysaccharide-preconditioned dental follicle cells promote periodontal regeneration in an inflammatory microenvironment. ACS Biomater Sci Eng. 2020;6:5797–810. 33320548 10.1021/acsbiomaterials.0c00882

[73] Sui B, Chen C, Kou X, Li B, Xuan K, Shi S, et al. Pulp stem cell–mediated functional pulp regeneration. J Dent Res. 2019;98:27–35. 30372659 10.1177/0022034518808754

[74] Kim SG, Malek M, Sigurdsson A, Lin LM, Kahler B. Regenerative endodontics: a comprehensive review. Int Endod J. 2018;51:1367–88. 29777616 10.1111/iej.12954

[75] Wu M, Liu X, Li Z, Huang X, Guo H, Guo X, et al. SHED aggregate exosomes shuttled miR-26a promote angiogenesis in pulp regeneration via TGF‐β/SMAD2/3 signalling. Cell Prolif. 2021;54:e13074. 34101281 10.1111/cpr.13074 PMC8249784

[76] Huang C-C, Narayanan R, Alapati S, Ravindran S. Exosomes as biomimetic tools for stem cell differentiation: applications in dental pulp tissue regeneration. Biomaterials. 2016;111:103–15. 27728810 10.1016/j.biomaterials.2016.09.029 PMC5293278

[77] Wang D, Lyu Y, Yang Y, Zhang S, Chen G, Pan J, et al. Schwann cell-derived EVs facilitate dental pulp regeneration through endogenous stem cell recruitment via SDF-1/CXCR4 axis. Acta Biomater. 2022;140:610–24. 34852303 10.1016/j.actbio.2021.11.039

[78] Zhang S, Yang Y, Jia S, Chen H, Duan Y, Li X, et al. Exosome-like vesicles derived from Hertwig’s epithelial root sheath cells promote the regeneration of dentin-pulp tissue. Theranostics. 2020;10:5914–31. 32483427 10.7150/thno.43156 PMC7254987

[79] Chen W-J, Xie J, Lin X, Ou M-H, Zhou J, Wei X-L, et al. The role of small extracellular vesicles derived from lipopolysaccharide-preconditioned human dental pulp stem cells in dental pulp regeneration. J Endod. 2021;47:961–9. 33775732 10.1016/j.joen.2021.03.010

[80] Chen Y, Ma Y, Yang X, Chen J, Yang B, Tian W. The application of pulp tissue derived-exosomes in pulp regeneration: a novel cell-homing approach. Int J Nanomed. 2022;17:465–76. 10.2147/IJN.S342685 PMC8809678 35125868

[81] Li Z, Wu M, Liu S, Liu X, Huan Y, Ye Q, et al. Apoptotic vesicles activate autophagy in recipient cells to induce angiogenesis and dental pulp regeneration. Mol Ther. 2022;30:3193–208. 35538661 10.1016/j.ymthe.2022.05.006 PMC9552912

[82] Gugliandolo A, Mazzon E. Dental mesenchymal stem cell secretome: an intriguing approach for neuroprotection and neuroregeneration. Int J Mol Sci. 2021;23:456. 35008878 10.3390/ijms23010456 PMC8745761

[83] Sharma V, Srinivasan A, Nikolajeff F, Kumar S. Biomineralization process in hard tissues: the interaction complexity within protein and inorganic counterparts. Acta Biomater. 2021;120:20–37. 32413577 10.1016/j.actbio.2020.04.049

[84] Pandya M, Rosene L, Farquharson C, Millán JL, Diekwisch TGH. Intravesicular phosphatase PHOSPHO1 function in enamel mineralization and prism formation. Front Physiol. 2017;8:805. 29089903 10.3389/fphys.2017.00805 PMC5651051

[85] Chen Y, Koshy R, Guirado E, George A. STIM1 a calcium sensor promotes the assembly of an ECM that contains extracellular vesicles and factors that modulate mineralization. Acta Biomater. 2021;120:224–39. 33130308 10.1016/j.actbio.2020.10.011 PMC7796999

[86] Swanson WB, Gong T, Zhang Z, Eberle M, Niemann D, Dong R, et al. Controlled release of odontogenic exosomes from a biodegradable vehicle mediates dentinogenesis as a novel biomimetic pulp capping therapy. J Controlled Release. 2020;324:679–94. 10.1016/j.jconrel.2020.06.006 PMC7429296 32534011

[87] Wen B, Huang Y, Qiu T, Huo F, Xie L, Liao L, et al. Reparative dentin formation by dentin matrix proteins and small extracellular vesicles. J Endod. 2021;47:253–62. 33245976 10.1016/j.joen.2020.11.017

[88] Zhao Y, Huang Y, Liu H, Tan K, Wang R, Jia L et al. Macrophages with different polarization phenotypes influence cementoblast mineralization through exosomes. Stem Cells Int. 2022;1:1–16. 10.1155/2022/4185972 PMC9507802 36159746

[89] Jiang N, Xiang L, He L, Yang G, Zheng J, Wang C, et al. Exosomes mediate epithelium–mesenchyme crosstalk in organ development. ACS Nano. 2017;11:7736–46. 28727410 10.1021/acsnano.7b01087 PMC5634743

[90] Tanaka E, del Pozo R, Tanaka M, Asai D, Hirose M, Iwabe T, et al. Three-dimensional finite element analysis of human temporomandibular joint with and without disc displacement during jaw opening. Med Eng Phys. 2004;26:503–11. 15234686 10.1016/j.medengphy.2004.03.001

[91] Lee Y-H, Park H-K, Auh Q-S, Nah H, Lee JS, Moon H-J, et al. Emerging potential of exosomes in regenerative medicine for temporomandibular joint osteoarthritis. Int J Mol Sci. 2020;21:1541. 32102392 10.3390/ijms21041541 PMC7073204

[92] Zhang S, Teo KYW, Chuah SJ, Lai RC, Lim SK, Toh WS. MSC exosomes alleviate temporomandibular joint osteoarthritis by attenuating inflammation and restoring matrix homeostasis. Biomaterials. 2019;200:35–47. 30771585 10.1016/j.biomaterials.2019.02.006

[93] Corrigendum to. ‘Exosomes derived from hypoxia preconditioned mesenchymal stem cells laden in a silk hydrogel promote cartilage regeneration via the miR-205–5p/PTEN/AKT pathway’ [Acta Biomaterialia 143 (2022) 173–188]. Acta Biomater. 2022;151:662–3. 10.1016/j.actbio.2022.02.026 35202856

[94] Luo P, Jiang C, Ji P, Wang M, Xu J. Exosomes of stem cells from human exfoliated deciduous teeth as an anti-inflammatory agent in temporomandibular joint chondrocytes via miR-100-5p/mTOR. Stem Cell Res Ther. 2019;10:1–12. 31358056 10.1186/s13287-019-1341-7 PMC6664713

[95] Zhang S, Chu WC, Lai RC, Lim SK, Hui JHP, Toh WS. Exosomes derived from human embryonic mesenchymal stem cells promote osteochondral regeneration. Osteoarthritis Cartilage. 2016;24:2135–40. 27390028 10.1016/j.joca.2016.06.022

[96] Diomede F, Fonticoli L, Marconi GD, Della Rocca Y, Rajan TS, Trubiani O, et al. Decellularized dental pulp, extracellular vesicles, and 5-azacytidine: a new tool for endodontic regeneration. Biomedicines. 2022;10:403. 35203612 10.3390/biomedicines10020403 PMC8962372

[97] Yang S, Liu Q, Chen S, Zhang F, Li Y, Fan W, et al. Extracellular vesicles delivering nuclear factor I/C for hard tissue engineering: treatment of apical periodontitis and dentin regeneration. J Tissue Eng. 2022;13:204173142210840. 10.1177/20417314221084095 PMC8935403 35321254

[98] Hu X, Zhong Y, Kong Y, Chen Y, Feng J, Zheng J. Lineage-specific exosomes promote the odontogenic differentiation of human dental pulp stem cells (DPSCs) through TGFβ1/smads signaling pathway via transfer of microRNAs. Stem Cell Res Ther. 2019;10:1–14. 31196201 10.1186/s13287-019-1278-x PMC6567518

[99] Yan C, Li N, Xiao T, Ye X, Fu L, Ye Y, et al. Extracellular vesicles from the inflammatory microenvironment regulate the osteogenic and odontogenic differentiation of periodontal ligament stem cells by miR-758-5p/LMBR1/BMP2/4 axis. J Transl Med. 2022;20:208. 35562763 10.1186/s12967-022-03412-9 PMC9103284

[100] Noronha-Matos JB, Correia-de-Sá P. Mesenchymal stem cells ageing: targeting the Purinome to promote osteogenic differentiation and bone repair. J Cell Physiol. 2016;231:1852–61. 26754327 10.1002/jcp.25303

[101] Čebatariūnienė A, Kriaučiūnaitė K, Prunskaitė J, Tunaitis V, Pivoriūnas A. Extracellular vesicles suppress basal and Lipopolysaccharide-Induced NFκB activity in human periodontal ligament stem cells. Stem Cells Dev. 2019;28:1037–49. 31017040 10.1089/scd.2019.0021

[102] Guo J, Zhou F, Liu Z, Cao Y, Zhao W, Zhang Z, et al. Exosome-shuttled mitochondrial transcription factor A mRNA promotes the osteogenesis of dental pulp stem cells through mitochondrial oxidative phosphorylation activation. Cell Prolif. 2022;55:e13324. 36054692 10.1111/cpr.13324 PMC9715363

[103] Lv P, Gao P, Tian G, Yang Y, Mo F, Wang Z, et al. Osteocyte-derived exosomes induced by mechanical strain promote human periodontal ligament stem cell proliferation and osteogenic differentiation via the miR-181b-5p/PTEN/AKT signaling pathway. Stem Cell Res Ther. 2020;11:1–15. 32680565 10.1186/s13287-020-01815-3 PMC7367226

[104] Wang J, Xia Y, Li J, Wang W. miR-129–5p in exosomes inhibits diabetes-associated osteogenesis in the jaw via targeting FZD4. Biochem Biophys Res Commun. 2021;566:87–93. 34119828 10.1016/j.bbrc.2021.05.072

[105] Shuo Y, Biao Z, Yu TX, Ying YH, Bo Q, Sheng ZL, et al. Exosomes derived from human umbilical cord mesenchymal stem cells enhance the osteoblastic differentiation of periodontal ligament stem cells under high glucose conditions through the PI3K/AKT signaling pathway. Biomed Env Sci. 2022;35:811–20. 36189996 10.3967/bes2022.105

[106] Orimo H. The mechanism of mineralization and the role of alkaline phosphatase in health and disease. J Nippon Med Sch. 2010;77:4–12. 20154452 10.1272/jnms.77.4

[107] Ansari S, de Wildt BWM, Vis MAM, de Korte CE, Ito K, Hofmann S, et al. Matrix vesicles: role in bone mineralization and potential use as therapeutics. Pharmaceuticals. 2021;14:289. 33805145 10.3390/ph14040289 PMC8064082

[108] Chaudhary SC, Kuzynski M, Bottini M, Beniash E, Dokland T, Mobley CG, et al. Phosphate induces formation of matrix vesicles during odontoblast-initiated mineralization in vitro. Matrix Biol. 2016;52–54:284–300. 26883946 10.1016/j.matbio.2016.02.003 PMC4875887

[109] Zhang Y, Song Y, Ravindran S, Gao Q, Huang CC, Ramachandran A, et al. DSPP contains an IRES element responsible for the translation of dentin phosphophoryn. J Dent Res. 2014;93:155–61. 24352500 10.1177/0022034513516631 PMC3895336

[110] Deng D-K. Roles of extracellular vesicles in periodontal homeostasis and their therapeutic potential. J Nanobiotechnol. 2022;20:545. 10.1186/s12951-022-01757-3 PMC9801622 36585740

[111] Zhang Z, Shuai Y, Zhou F, Yin J, Hu J, Guo S, et al. PDLSCs regulate angiogenesis of periodontal ligaments via VEGF transferred by exosomes in periodontitis. Int J Med Sci. 2020;17:558–67. 32210705 10.7150/ijms.40918 PMC7085218

[112] Zhang S, Thiebes AL, Kreimendahl F, Ruetten S, Buhl EM, Wolf M, et al. Extracellular vesicles-loaded fibrin gel supports rapid neovascularization for dental pulp regeneration. Int J Mol Sci. 2020;21:4226. 32545804 10.3390/ijms21124226 PMC7352754

[113] Liu P, Qin L, Liu C, Mi J, Zhang Q, Wang S, et al. Exosomes derived from hypoxia-conditioned stem cells of human deciduous exfoliated teeth enhance angiogenesis via the transfer of let-7f-5p and miR-210-3p. Front Cell Dev Biol. 2022;10:879877. 35557954 10.3389/fcell.2022.879877 PMC9086315

[114] Xian X, Gong Q, Li C, Guo B, Jiang H. Exosomes with highly angiogenic potential for possible use in pulp regeneration. J Endod. 2018;44:751–8. 29426641 10.1016/j.joen.2017.12.024

[115] Barnabei L, Laplantine E, Mbongo W, Rieux-Laucat F, Weil R. NF-κB: at the borders of autoimmunity and inflammation. Front Immunol. 2021;12:716469. 34434197 10.3389/fimmu.2021.716469 PMC8381650

[116] Wang Z, Maruyama K, Sakisaka Y, Suzuki S, Tada H, Suto M, et al. Cyclic stretch force induces periodontal ligament cells to secrete exosomes that suppress IL-1β production through the inhibition of the NF-κB signaling pathway in macrophages. Front Immunol. 2019;10:1310. 31281309 10.3389/fimmu.2019.01310 PMC6595474

[117] Sun J, Hu Y, Fu Y, Zou D, Lu J, Lyu C. Emerging roles of platelet concentrates and platelet-derived extracellular vesicles in regenerative periodontology and implant dentistry. APL Bioeng. 2022;6:031503. 36061076 10.1063/5.0099872 PMC9439711

[118] Zheng J, Kong Y, Hu X, Li Z, Li Y, Zhong Y, et al. MicroRNA-enriched small extracellular vesicles possess odonto-immunomodulatory properties for modulating the immune response of macrophages and promoting odontogenesis. Stem Cell Res Ther. 2020;11:1–14. 33256846 10.1186/s13287-020-02039-1 PMC7708107

[119] Yasuda H. Discovery of the RANKL/RANK/OPG system. J Bone Min Metab. 2021;39:2–11. 10.1007/s00774-020-01175-1 33389131

[120] Yan J, Luo M, Chen Z, He B. The Function and Role of the Th17/Treg cell balance in inflammatory bowel disease. J Immunol Res. 2020;1:8813558. 10.1155/2020/8813558 PMC7755495 33381606

[121] Yan H-C, Yu T-T, Li J, Qiao Y-Q, Wang L-C, Zhang T, et al. The delivery of extracellular vesicles loaded in biomaterial scaffolds for bone regeneration. Front Bioeng Biotechnol. 2020;8:1015. 32974327 10.3389/fbioe.2020.01015 PMC7466762

[122] Ju Y, Hu Y, Yang P, Xie X, Fang B. Extracellular vesicle-loaded hydrogels for tissue repair and regeneration. Mater Today Bio. 2023;18:100522. 36593913 10.1016/j.mtbio.2022.100522 PMC9803958

[123] Suchanek W, Yoshimura M. Processing and properties of hydroxyapatite-based biomaterials for use as hard tissue replacement implants. J Mater Res. 1998;13:94–117.

[124] Kong H, Liu P, Li H, Zeng X, Xu P, Yao X, et al. Mesenchymal stem cell-derived extracellular vesicles: the novel therapeutic option for regenerative dentistry. Stem Cell Rev Rep. 2023;19:46–58. 35132538 10.1007/s12015-022-10342-y

[125] Lv L, Sheng C, Zhou Y. Extracellular vesicles as a novel therapeutic tool for cell-free regenerative medicine in oral rehabilitation. J Oral Rehabil. 2020;47:29–54. 31520537 10.1111/joor.12885

[126] Liu Z, Wang S, Huo N, Yang S, Shi Q, Xu J. Extracellular vesicles: a potential future strategy for dental and maxillofacial tissue repair and regeneration. Front Physiol. 2022;13:1012241. 36479350 10.3389/fphys.2022.1012241 PMC9719951

[127] Liu M, Liu X, Su Y, Li S, Chen Y, Liu A, et al. Emerging role of mesenchymal stem cell-derived extracellular vesicles in oral and craniomaxillofacial tissue regenerative medicine. Front Bioeng Biotechnol. 2022;10:1054370. 36524049 10.3389/fbioe.2022.1054370 PMC9744765

[128] Li Q, Yu H, Sun M, Yang P, Hu X, Ao Y, et al. The tissue origin effect of extracellular vesicles on cartilage and bone regeneration. Acta Biomater. 2021;125:253–66. 33657452 10.1016/j.actbio.2021.02.039

[129] Fafián-Labora J, Lesende-Rodriguez I, Fernández-Pernas P, Sangiao-Alvarellos S, Monserrat L, Arntz OJ, et al. Effect of age on pro-inflammatory miRNAs contained in mesenchymal stem cell-derived extracellular vesicles. Sci Rep. 2017;7:43923. 28262816 10.1038/srep43923 PMC5338265

[130] Xia Y, He X-T, Xu X-Y, Tian B-M, An Y, Chen F-M. Exosomes derived from M0, M1 and M2 macrophages exert distinct influences on the proliferation and differentiation of mesenchymal stem cells. PeerJ. 2020;8:e8970. 32355576 10.7717/peerj.8970 PMC7185029

[131] Monguió-Tortajada M, Gálvez-Montón C, Bayes-Genis A, Roura S, Borràs FE. Extracellular vesicle isolation methods: rising impact of size-exclusion chromatography. Cell Mol Life Sci. 2019;76:2369–82. 30891621 10.1007/s00018-019-03071-y PMC11105396

[132] Xu R, Greening DW, Zhu H-J, Takahashi N, Simpson RJ. Extracellular vesicle isolation and characterization: toward clinical application. J Clin Invest. 2016;126:1152–62. 27035807 10.1172/JCI81129 PMC4811150

[133] Nath Neerukonda S, Egan NA, Patria J, Assakhi I, Tavlarides-Hontz P, Modla S, et al. Comparison of exosomes purified via ultracentrifugation (UC) and total exosome isolation (TEI) reagent from the serum of Marek’s disease virus (MDV)-vaccinated and tumor-bearing chickens. J Virol Methods. 2019;263:1–9. 30316797 10.1016/j.jviromet.2018.10.004

[134] Lin Y, Anderson JD, Rahnama LMA, Gu SV, Knowlton AA. Exosomes in disease and regeneration: biological functions, diagnostics, and beneficial effects. Am J Physiol-Heart Circ Physiol. 2020;319:H1162–80. 32986962 10.1152/ajpheart.00075.2020 PMC7792703

[135] Zhao J, Ding Y, He R, Huang K, Liu L, Jiang C, et al. Dose-effect relationship and molecular mechanism by which BMSC-derived exosomes promote peripheral nerve regeneration after crush injury. Stem Cell Res Ther. 2020;11:1–17. 32811548 10.1186/s13287-020-01872-8 PMC7437056

[136] Chen Y-S, Lin E-Y, Chiou T-W, Harn H-J. Exosomes in clinical trial and their production in compliance with good manufacturing practice. Tzu Chi Med J. 2020;32:113. 10.4103/tcmj.tcmj_182_19 PMC7137364 32269942

[137] Chen A, Tian H, Yang N, Zhang Z, Yang G-Y, Cui W, et al. Towards extracellular vesicle delivery systems for tissue regeneration: material design at the molecular level. Extracell Vesicles Circ Nucl Acids. 2022;3:323–56. 10.20517/evcna.2022.37 PMC11648451 39697358

[138] Gao Y, Yuan Z, Yuan X, Wan Z, Yu Y, Zhan Q, et al. Bioinspired porous microspheres for sustained hypoxic exosomes release and vascularized bone regeneration. Bioact Mater. 2022;14:377–88. 35386817 10.1016/j.bioactmat.2022.01.041 PMC8964815

[139] Xie L, Guan Z, Zhang M, Lyu S, Thuaksuban N, Kamolmattayakul S et al. Exosomal circLPAR1 promoted osteogenic differentiation of Homotypic Dental Pulp Stem cells by competitively binding to hsa-miR-31. BioMed Res Int. 2020;1:1–13. 10.1155/2020/6319395 PMC7539105 33062690

[140] Sun J, Wang Z, Liu P, Hu Y, Li T, Yang J, et al. Exosomes derived from human gingival mesenchymal stem cells attenuate the inflammatory response in periodontal ligament stem cells. Front Chem. 2022;10:863364. 35464198 10.3389/fchem.2022.863364 PMC9019468

