# Supplementary material for: Extracellular vesicles as therapeutic tools in regenerative dentistry
Source: Stem Cell Res Ther. 2024 Oct 14;15:365. doi: 10.1186/s13287-024-03936-5 (PMC11476107; doi:10.1186/s13287-024-03936-5)

# Supplementary Attachments

Link and Copyright license of figures reused in Figure 4 and 6.

- **Figure 4 (a)**

Link of original article <https://doi.org/10.1021/acsbiomaterials.0c00882>

Figures be reused: Figure 5 A and D, Figure 6 B

Copyright license: <https://creativecommons.org/licenses/by-nc/2.5/cn/legalcode.zh-hans>

- **Figure 4 (b)**

Link of original article <https://doi.org/10.1177/0022034520906793>

Figures be reused: Figure 2 B, C and D

Copyright license:


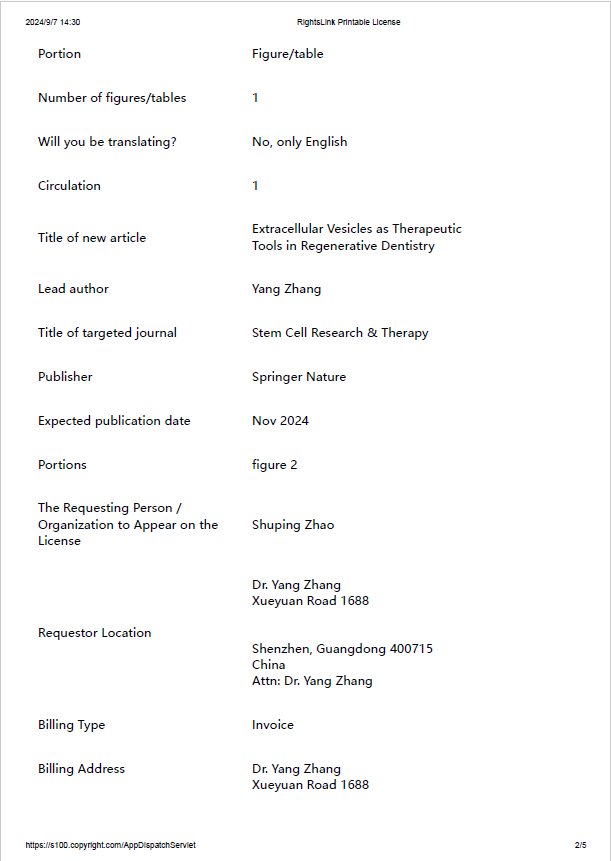

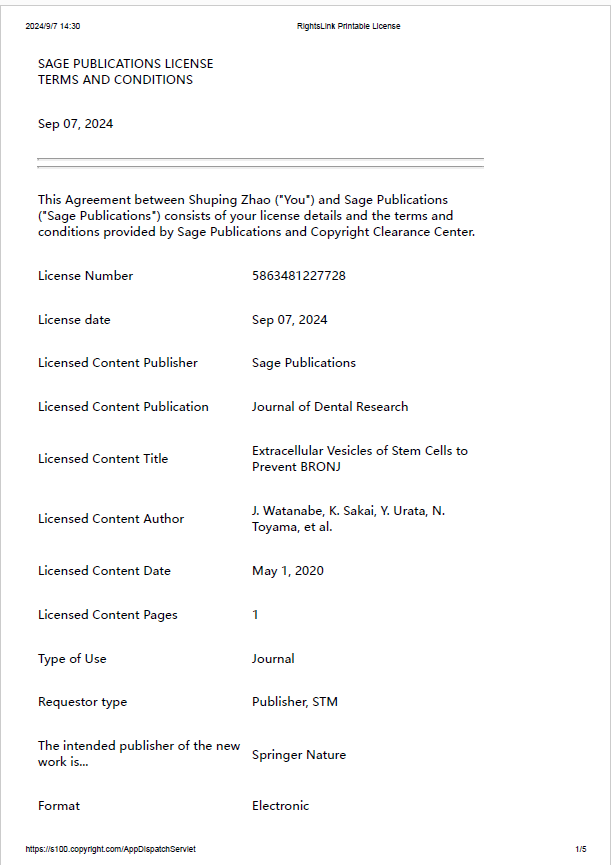


- **Figure 4 (c)**

Link of original article <https://doi.org/10.1016/j.joen.2020.11.017>

Figures be reused: Figure 4 A, B and C

Copyright license:

**
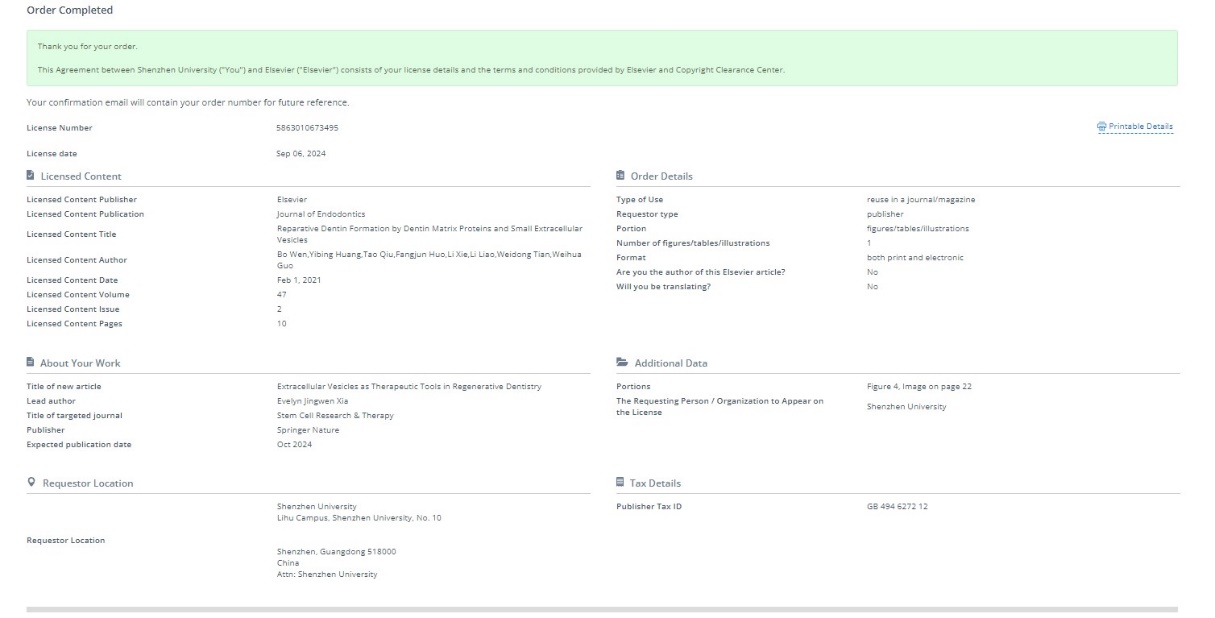
**

- **Figure 4 (d)**

Link of original article <https://doi.org/10.1016/j.biomaterials.2023.122367>

Figure be reused: Figure 2D


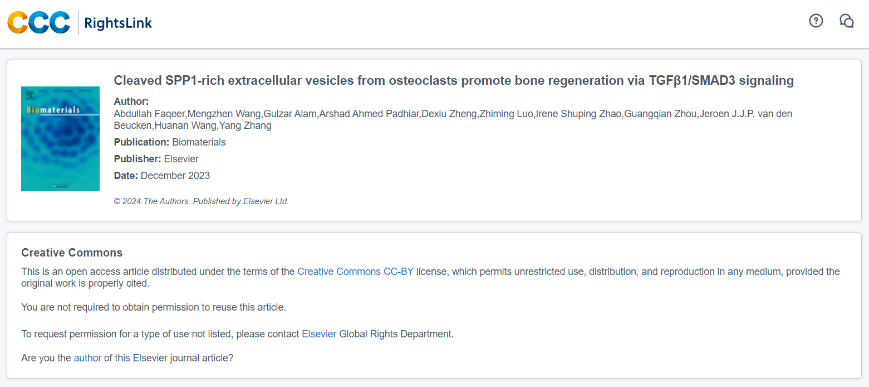
Copyright license:

- **Figure 6 (a)**

Link of original article <https://doi.org/10.1016/j.ymthe.2022.05.006>

Figure be reused: Figure 8


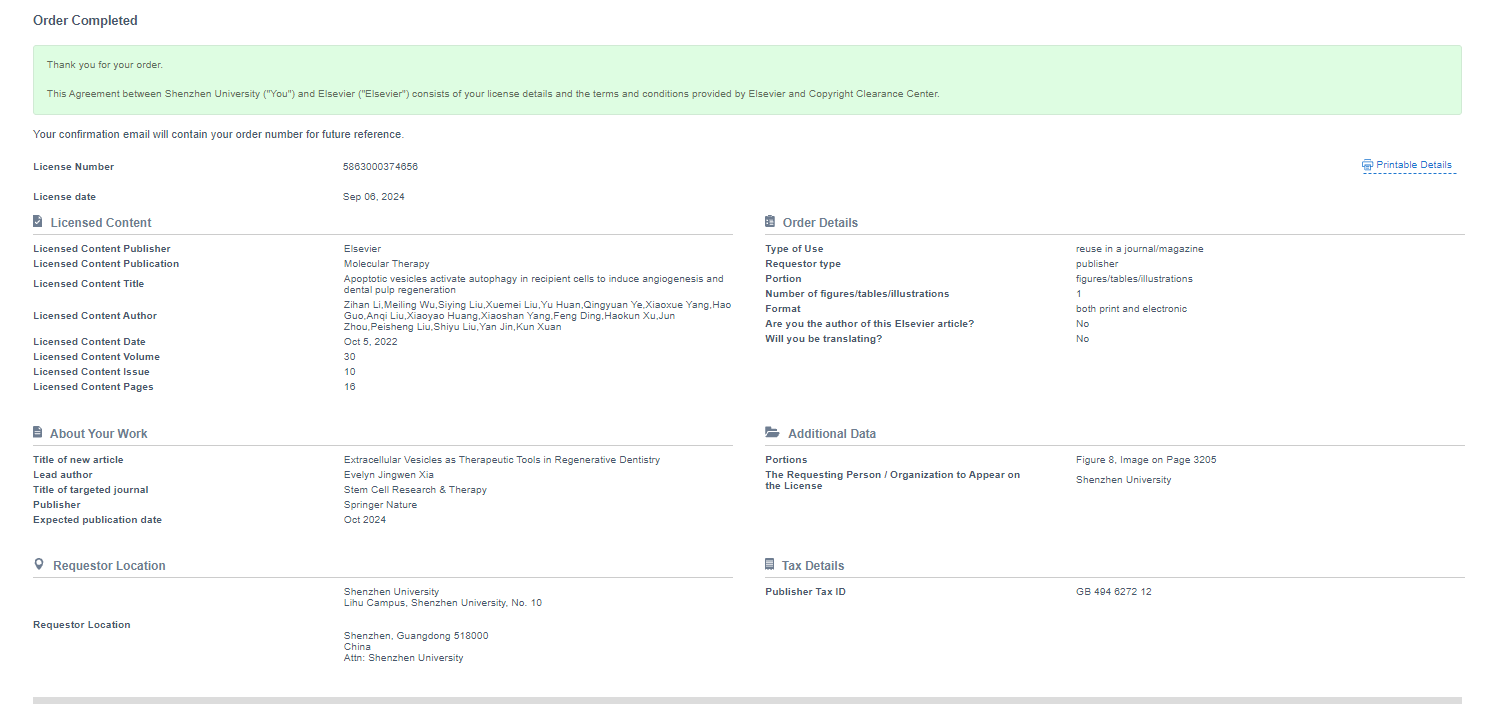
Copyright license:

- **Figure 6 (b)**

Link of original article <https://doi.org/10.1186/s12967-022-03412-9>

Figure be reused: Figure 8

Copyright license:


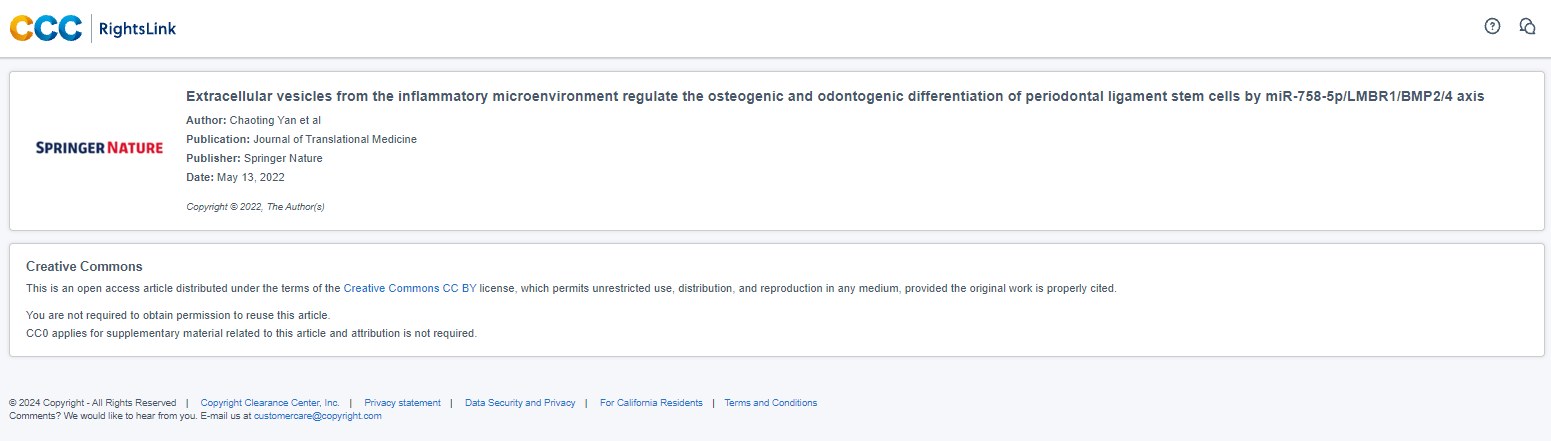


- **Figure 6 (c)**

Link of original article <https://doi.org/10.1016/j.actbio.2020.12.046>

Figure be reused: Schema 1

Copyright license:


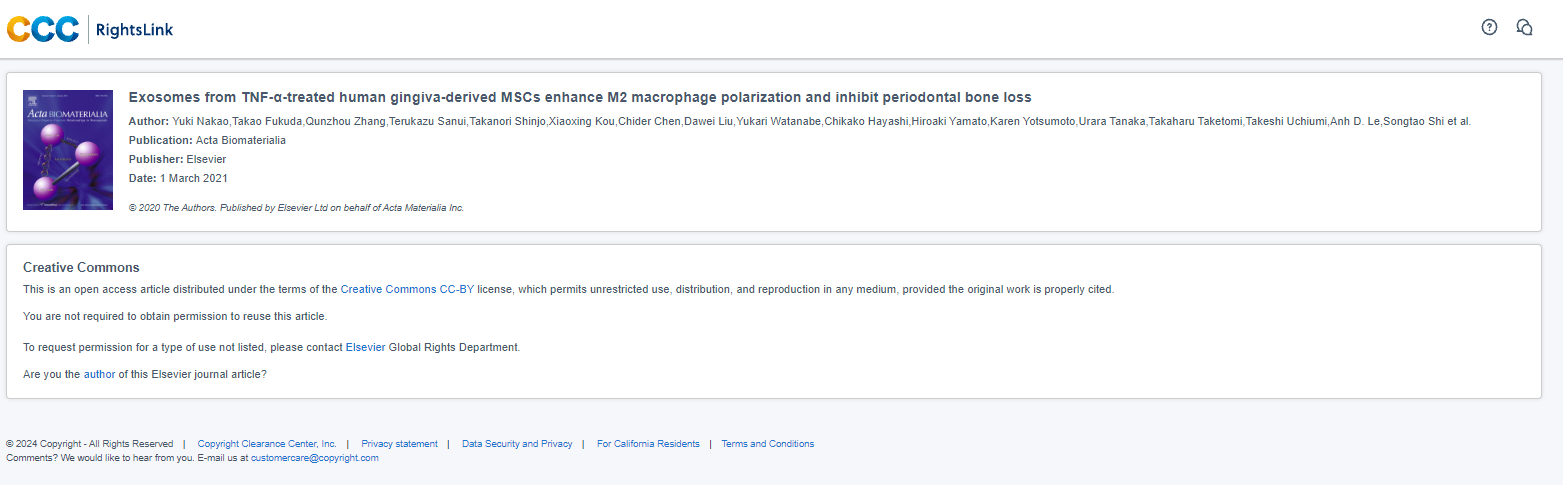

Supplement: Supplementary file 1 — Supplementary material 1 [file 13287_2024_3936_MOESM1_ESM.docx]
